# Graphene Oxide and Reduced Derivatives, as Powder or Film Scaffolds, Differentially Promote Dopaminergic Neuron Differentiation and Survival

**DOI:** 10.3389/fnins.2020.570409

**Published:** 2020-12-21

**Authors:** Noela Rodriguez-Losada, Rune Wendelbob, M. Carmen Ocaña, Amelia Diaz Casares, Roberto Guzman de Villoría, Jose A. Aguirre Gomez, Miguel A. Arraez, Pedro Gonzalez-Alegre, Miguel A. Medina, Ernest Arenas, Jose A. Narvaez

**Affiliations:** ^1^Department Human Physiology, Faculty of Medicine, Biomedicine Research Institute of Malaga (IBIMA C07), University of Malaga, Malaga, Spain; ^2^Department of Didactic Science Education, Faculty of Science Education, University of Malaga, Malaga, Spain; ^3^ABALONYX AS., Oslo, Norway; ^4^Department of Molecular Biology and Biochemistry, Faculty of Sciences, and IBIMA (Biomedical Research Institute of Málaga), Andalucía Tech, University of Málaga, Málaga, Spain; ^5^CIBER de Enfermedades Raras (CIBERER), Málaga, Spain; ^6^Laboratory of Mechanical Engineering Applied to Design, Manufacturing and Applications of Composite Materials (LAMCOM), Department of Mechanical Engineering, University of Salamanca, Escuela Politécnica Superior de Zamora, Zamora, Spain; ^7^Neurosurgery Unit, Department Neurosurgery, Biomedicine Research Institute of Malaga (IBIMA), Hospital Regional de Malaga, Andalusian Health System (SAS), Malaga, Spain; ^8^Raymond G. Perelman Center for Cellular & Molecular Therapeutics, The Children's Hospital of Philadelphia, Philadelphia, PA, United States; ^9^Department of Neurology, Perelman School of Medicine at the University of Pennsylvania, Philadelphia, PA, United States; ^10^Laboratory of Molecular Neurobiology, Department of Medical Biochemistry and Biophysics, Karolinska Institute, Stockholm, Sweden

**Keywords:** Parkinson's disease, bioenergetic dysfunction, neurodifferentiation, neuronal dysfunction, graphene oxide

## Abstract

Emerging scaffold structures made of carbon nanomaterials, such as graphene oxide (GO) have shown efficient bioconjugation with common biomolecules. Previous studies described that GO promotes the differentiation of neural stem cells and may be useful for neural regeneration. In this study, we examined the capacity of GO, full reduced (FRGO), and partially reduced (PRGO) powder and film to support survival, proliferation, differentiation, maturation, and bioenergetic function of a dopaminergic (DA) cell line derived from the mouse substantia nigra (SN4741). Our results show that the morphology of the film and the species of graphene (GO, PRGO, or FRGO) influences the behavior and function of these neurons. In general, we found better biocompatibility of the film species than that of the powder. Analysis of cell viability and cytotoxicity showed good cell survival, a lack of cell death in all GO forms and its derivatives, a decreased proliferation, and increased differentiation over time. Neuronal maturation of SN4741 in all GO forms, and its derivatives were assessed by increased protein levels of tyrosine hydroxylase (TH), dopamine transporter (DAT), the glutamate inward rectifying potassium channel 2 (GIRK2), and of synaptic proteins, such as synaptobrevin and synaptophysin. Notably, PRGO-film increased the levels of Tuj1 and the expression of transcription factors specific for midbrain DA neurons, such as Pitx3, Lmx1a, and Lmx1b. Bioenergetics and mitochondrial dysfunction were evaluated by measuring oxygen consumption modified by distinct GO species and were different between powder and film for the same GO species. Our results indicate that PRGO-film was the best GO species at maintaining mitochondrial function compared to control. Finally, different GO forms, and particularly PRGO-film was also found to prevent the loss of DA cells and the decrease of the α-synuclein (α-syn) in a molecular environment where oxidative stress has been induced to model Parkinson's disease. In conclusion, PRGO-film is the most efficient graphene species at promoting DA differentiation and preventing DA cell loss, thus becoming a suitable scaffold to test new drugs or develop constructs for Parkinson's disease cell replacement therapy.

## Introduction

Over the last decade, it has become increasingly apparent that it is essential to provide cells with appropriate physical substrates to allow regeneration. Scaffolds aid in supporting cellular architecture and may also regulate processes, such as cell polarization and differentiation, thus contributing to regeneration. Many types of materials have been developed as cellular matrices, such as graphene, discovered by Novoselov and Geim (Novoselov et al., 2004). Graphene has recently emerged as a reliable material to create scaffolds for the neural tissue (Fabbro et al., 2016) because of its biocompatibility and electroconductive and physicochemical properties. Graphene is a 2-dimensional material consisting of rings of carbon atoms with a hexagonal lattice structure (Ryu and Kim, 2013) with an excellent electrical conductivity originated in the sp^2^ hybridized carbons network (Hess et al., 2013). It is also known that graphene's electric conductivity improves the neuronal differentiation of neural stem cells (Park et al., 2011). In foam form, graphene has inherent mechanical properties and capability for adsorption of proteins and substances with low molecular weight (Yavari et al., 2011), which facilitates cellular interactions as well as cell differentiation and proliferation (Lim et al., 2011). It has also been described that graphene, and especially graphene oxide (GO), has an individual capacity to promote neurite outgrowth in cultured cells (Li et al., 2011). At the same time, graphene has been shown to activate apoptosis in a glioblastoma cell line (U118), suggesting that it does not support tumor growth and that its use is safe (Jaworski et al., 2013).

In this study, we evaluated whether GO and its derivatives (GOd), partial reduced GO (PRGO) and fully reduced GO (FRGO), as powder or film (micro flakes), can promote neuronal differentiation, maturation and neuroprotection in a substantia nigra dopaminergic (DA) cell line (SN4741) (Son et al., 1999). We found that different types of GO induce specific changes in the metabolism, the capacity of DA cells to proliferate and differentiate, as well as their responses to DA neurotoxins, such as a Rot, used to model the oxidative stress in Parkinson's disease (Sherer et al., 2003). Our results provide critical information to design appropriate scaffolds for future application in regenerative medicine, aiming to replace substantia nigra DA neurons, the principal cell type affected in Parkinson's disease. In this study we evaluated whether GO and its derivatives (GOd), partial reduced GO (PRGO) and fully reduced GO (FRGO), as powder or film (micro flakes), can promote neuronal differentiation, maturation and neuroprotection in a substantia nigra dopaminergic (DA) cell line (SN4741) (Son et al., 1999).

## Materials and Methods

### Graphene Derivative Production

**Figure 1:**
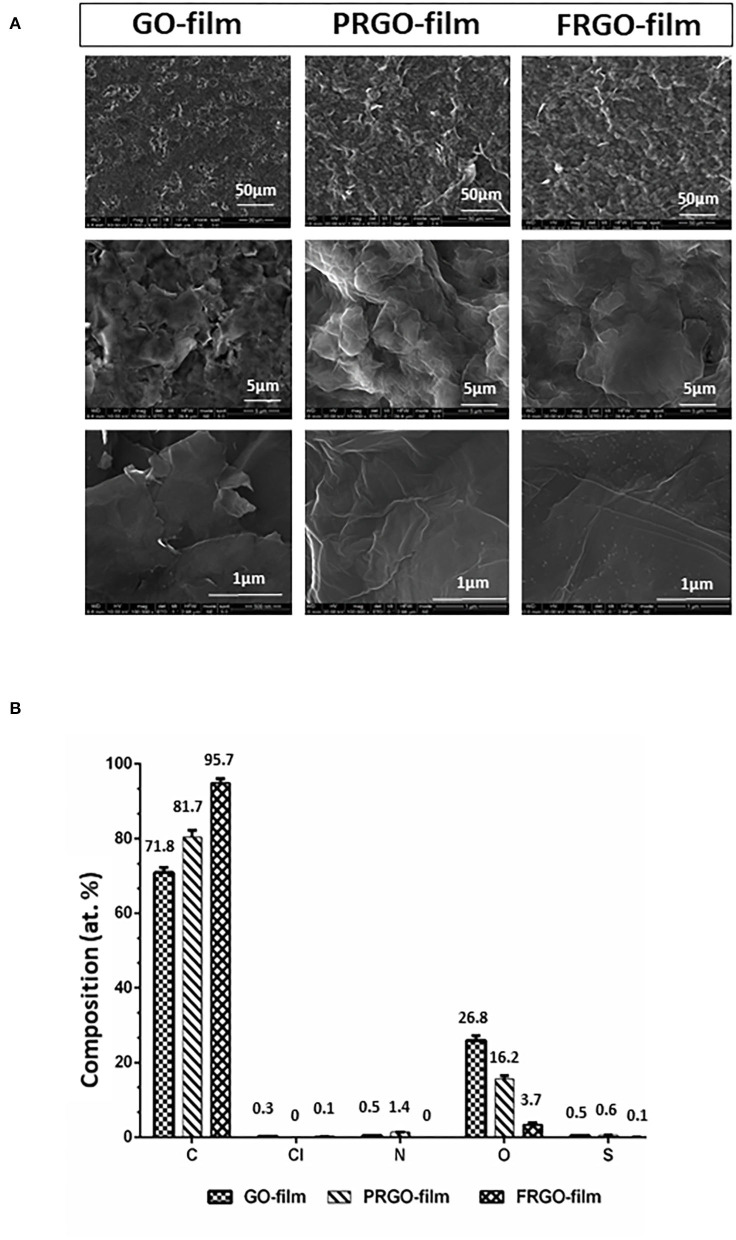
Structure and composition of different GO derivatives (GOd). **(A)** Representative pictures of conventional and environmental Scanning Electron Microscopy micrographs of GO film, partially reduced GO (PRGO) film and fully reduced GO (FRGO) film showing surface texture at different magnifications (1,000×, 10,000×, and 100,000×). **(B)** Chemical composition measured by XPS in atomic concentrations (atom: at. %). Graphs show two measures and their standard deviation.

GO was prepared by Abalonyx AS (Oslo, Norway) from natural graphite powder following a modification of the “Hummers method” (Hummers and Offeman, 1958) by using potassium permanganate as the oxidation agent. The prepared GO was washed with HCl to avoid precipitation of manganese as MnO_2_ and subsequently centrifuged to form an aqueous paste. The paste was mixed with particles of NaCl, dried in the form of films, and the subsequent dissolution of NaCl produced a GO scaffold. The films were either used without further heat treatment to obtain GO-film or heated to 300°C in air to obtain partly (PRGO) or to 1,100°C in air to obtain fully reduced GO (FRGO) in the form of powder or film. Two crucial differences between virgin and reduced GO are that virgin GO is hydrophilic and non-conductive, whereas reduced GO (PRGO and FRGO) is hydrophobic and conductive. Samples of the films were analyzed for chemical composition using an X-Ray Photoelectron Spectroscopy analysis at high vacuum and conventional and environmental Scanning Electron Microscopy as used for the structure analysis at INA (Zaragoza, Spain). Chemical compositions are shown in Figure 1B.

### Treatment of SN4741 Cells With GO, PRGO, FRGO Powder and Film (Microflakes)

For morphological analysis, SN4741cells (Son et al., 1999) were cultivated at a density of 5.3 × 10^3^ cells/cm^2^ (48 well, Thermo Fisher Scientific Inc) and 2.6 × 10^3^ cells/cm^2^ (on top of the films) in DMEM supplemented with 10% FCS, glucose (0.6%), penicillin-streptomycin (50 U/ml), and L-glutamine (2 mM) at 5% CO_2_ humidified atmosphere as previously described (Son et al., 1999). Cells in culture were exposed to GO and derivatives, either powder (GO-powder, PRGO-powder, FRGO-powder) or film (GO-film, PRGO-film, FRGO-film). The films were used in the form of microflakes ranging up to 5–10 μm^2^. The capacity of cells to grow on the substrates was examined for up to 6 weeks in culture by fluorescence microscopy. Hoechst 33342 (Sigma-Aldrich) was used to label the nuclei and visualize the presence and distribution of cells on GO, PRGO, and FRGO scaffolds since they are opaque. Morphological changes and cytoarchitecture were followed using an inverted microscopy TE2000-U coupled to a camera Nikon DS5MC and a microscope Zeiss LSM700. Photographs were taken at the indicated time-points and conditions. The morphological changes were compared to SN4741 cells cultured in the same conditions, but the absence of GO.

### Analysis of Cellular Viability

#### MTT Assay

The viability of SN4741 cells seeded at an 11.75 × 10^5^ cells/cm^2^ for 3 days or 6.7 × 10^4^ cells/cm^2^ for 7 and 3.3 × 10^3^ cells/cm^2^ 15 days, respectively, and exposed to different types of GOd was analyzed. The experiments were conducted as previously described (Rodriguez-Losada et al., 2017). Negative controls (C–) correspond to cells cultured without GO and derivatives; positive controls (C+), were treated with 10% Triton X-100. Treatments were applied in triplicate and consisted of the addition of GO, PRGO, and FRGO in powder or film (microflakes) at concentrations of 0 (C–, also represented as a black line in the graphics used as a normalization data), 1, 5, 10, 20, 50, 100, 500, or 1,000 μg/ml in essential medium (DMEM) without phenolphthalein in normal conditions. Both powders and films (as microflakes) were suspended in DMEM at the stock concentration (2 mg/ml).

#### Live/Dead Assay

The LIVE/DEAD® Viability/Cytotoxicity Assay Kit (Invitrogen) was used to discriminate between live cells (viable cells) and dead cells (cells with damaged membranes, including apoptotic and necrotic signs). This assay can determine the live cells by staining them with calcein-AM (green stained) simultaneously with staining of dead cells using red Ethidium homodimer-1, which indicates loss of membrane integrity. SN4741 cells were cultured at a density of 2.4 × 10^4^ cells/cm^2^) with GO and derivatives, and after 7 days of culture, cells were processed following the manufacturer's recommendations, previously described (Rodriguez-Losada et al., 2017). The concentrations of GOd were 0 (negative control, C–), 5, 10, 20, and 50 μg/ml. The positive control (C+) consisted of cells treated with 10% Triton X-100. Stained cells were counted by flow cytometry using a BD Accuri™ software for analysis.

### Bioenergetics Dysfunction: OCR Measurement

Oxygen consumption rate (OCR) was measured using a Seahorse XF^e^24 Extracellular Analyzer (Agilent). SN4741 cells were treated with GO and derivatives (50 μg/ml) for 7 days and then seeded at a density of 1.8 × 10^4^ cells/cm^2^ on Seahorse XF^e^24 Cell Culture Microplates (Agilent). After additional overnight incubation, cells were washed twice with XF Base Medium (Agilent) containing 25 mM glucose, 4 mM glutamine, and 1 mM sodium pyruvate and incubated for an additional hour at 37°C in the absence of CO_2_ before the start of the assay. Four basal measurements were performed before the addition of 1 μM oligomycin, 0.6 μM FCCP and 1 μM antimycin-A, 1 μM Rotenone (Rot), with three measures between different injections. Each measurement cycle consisted of a mixing time of 3 min, a waiting time of 2 min, and a data acquisition period of 3 min. Data were normalized to the protein content of cells obtained by Bradford assay (Bio-Rad).

### Cell Cycle Study

#### Propidium Iodide (PI) Assay

SN4741 cells were seeded at an initial density of 1 × 10^4^ cells/cm^2^ for treatment at 3 and 2.4 × 10^3^ cells/cm^2^ for 5 and 7 days. Then at the end of the incubation of the treatments, the cells were trypsinized, centrifuged for 5 min at 800 rpm, suspended in PBS, washed and fixed in 66% ethanol for 2 h in ice. The pellet was centrifuged and suspended in PBS, then washed and incubated in 0.25% Triton X-100 for 30 min. Cells were stained in 500 μL PI (50 μg/ml in PBS) and RNase (100 μg/ml in PBS) for 30 min at 37°C in the darkness. The reaction was read with a Becton Dickenson FACsort channel FL2 (laser excitation 488 nm), and the cell cycle phase distribution was determined by an analytical DNA flow cytometer using Summit v4.3.02 software Dako cytomation.

#### Bromodeoxyuridine (BrdU) Assay

The cells were culture at a density of 1.3 × 10^4^ cells/cm^2^ for 3 days 4 × 10^3^ cells/cm^2^ for 5 and 7 days, on 13 mm slides in the absence or presence of graphene (GO; PRGO; FRGO in powder and film species) at 50 μg/ml. Then they were treated with 10 μM BrdU for 4 h. After the incubations, cells were fixed using 4% paraformaldehyde for 20 min, washed with PBS, treated with 70% ethanol for 20 min and with 4 N HCl for 2 min. Cells were blocked using 2% horse serum. The primary antibody anti-BrdU (Sigma-Aldrich) was incubated at 1:75 for 2 h at room temperature (RT). Then the cells were washed 3 times with PBS for 10 min. The secondary antibody anti-mouse FITC was used at a concentration of at 1: 500 dilution.

#### Western-Blot Analysis of Proteins Directly Involved in the Cell Cycle: Proliferation, Quiescence, and Senescence Processes

Controls and SN4741 cells were treated with GOd (at a concentration of 50 μg/ml) and grown in 90 mm well plates for 3 and 7 days at a density of 5.3 × 10^3^ cells/cm^2^ and 2.4 × 10^3^ cells/cm^2^, respectively. Cells were rinsed twice with PBS 1× and lysed using 100 μL of RIPA buffer containing proteases inhibitors (1% NP-40, 0.5% sodium deoxycholate, 0.1% SDS, 100 μg/ml PMSF, 30 μl/ml aprotinin, and 1 mM sodium orthovanadate). Lysates were scraped off, transferred to microcentrifuge tubes, passed through a 21-gauge needle, and centrifuged at 16,000 rpm for 20 min at 4°C. Lysates (20 μg of the cells) were heated at 95°C for 5 min in Laemmli buffer 1× and then analyzed by sodium dodecyl sulfate-polyacrylamide gel electrophoresis (SDS-PAGE, TGXTM Staining-Free Fast CastTM, Biorad) 10% at 15 V/cm for 1 h. Proteins were transferred to a PVDF membrane for immunoblotting and incubated for 4 h at RT in the blocking buffer containing 5% non-fat dry milk in TBS buffer (0.1% Tween-20 in 0.1% TBS). The primary antibody against P27-Kip (27 kDa, Abcam) was incubated at 1:5,000 at RT for 2 h. Primary antibodies against cyclin E (50 kDa, Abcam) cell cycle initiator, PH3-Ser10 (20 kDa, Abcam) specific marker to S/G2 phase transition; KI67 (Millipore, 360 kDa) specific marker to mitosis G2 phase no expressed in G0 phase, PCNA (36 kDa, Abcam), expressed in G2/M phase and SP30 (30 kDa, Santa Cruz), involved in senescent processes, were incubated overnight at 1:1,000 at 4°C. β-actin (42 kDa, Sigma) was used for the normalization of the total protein amount. Protein detection was performed with the corresponding secondary antibodies (1:10,000) diluted in the blocking buffer. Signals were detected with ECL systems (Super Signal West Dura Extended, Thermo-Fisher, Rockford, USA). Images and quantification of bands were analyzed using Image Lab 4.0.1 and the Fiji/ImageJ software in three different experiments.

### Dopaminergic Maturation

#### Immunostaining Assay

SN4741 cells (at a density of 2.4 × 10^3^ cells/cm^2^) treated with GOd for 21 days and control untreated cells were fixed at *in vitro* day 7 with 4% paraformaldehyde (w/v) in 0.1 M PBS, pH 7.4, for 20 min, rinsed three times with PBS and permeabilized with 0.1% Triton X-100 in PBS. Cells were blocked with 5% normal goat serum (NGS, Invitrogen) in PBS with 3% BSA (Invitrogen). Primary antibodies were used at 1:100 in PBS with 3% BSA overnight at 4°C. The following antibodies were used: rabbit anti-tyrosine hydroxylase (TH, Nobus biologicals); mouse anti-Tuj-1 (β-III tubulin 1, Promega); goat anti-GIRK2 (G-protein-regulated inward-rectifier potassium channel 2 protein, Abcam); rat anti-DAT (transmembrane DA transporter, Millipore). After incubation, cells were washed in PBS with 0.1% Tween-20 for 1 h at RT. Secondary antibodies (Alexa Fluor 488 goat anti-mouse or Alexa Fluor 555 goat anti-rabbit) were incubated for 2 h at 1:500 at RT in PBS with 3% BSA. Cells were washed for 1 h in PBS with 0.1% Tween-20, and afterwards, cells were stained with DAPI (nuclei marker, Sigma) at 1:200 for 15 min at RT. The analysis of the colocalization coefficient for fluorescence microscopy for quantifying protein interactions was performed using Pearson's correlation coefficient (PCC) as previously described using the software Fiji-ImageJ plugin Col2 (Dunn et al., 2011). The region of interest (ROI) for colocalization analysis was used after a deconvolution process for reducing the background (Landmann, 2002) of all confocal pictures (Zeiss Confocal). To minimize the randomized background, we assessed the Costes coefficient in all analyses (Colo2 plugin) to confirm that the correlation between proteins did not depend on artifactual effects. This statistical algorithm allows determining whether the colocalization is produced in one specific compartment or is random.

#### Immunoblotting for Dopaminergic Differentiation

SN4741 cells at a density of 2.4 × 10^3^ cells/cm^2^ were treated with GOd at a concentration of 50 μg/ml for the long term in culture (21 days) and grown in 60 mm well plates. Ten percent of the cell media was replaced each week to avoid interference with the GOd culture process, and changes in the concentrations since GOd as microflakes kept attached to the cells. Proteins detection was performed with primary antibodies diluted in blocking buffer: anti-TH (60 kDa; Nobus biologicals), anti-DAT (80 kDa; clone DAT-Nt, Millipore), anti-synaptobrevin (13 kDa; clone 69.1, Synaptic Systems Göttingen, Germany), anti-synaptophysin (38 kDa; SYP, clone SY38, Chemicon), and anti-GIRK2 (48 kDa; Nobus biologicals). The primary antibodies (1:5,000) were incubated at 4°C overnight in a shaker, and later the corresponding horse-radish peroxidase-conjugated (1:10,000) secondary antibody diluted in blocking buffer were added. Signals were detected with ECL systems (Super Signal West Dura Extended, Thermo-Fisher, Rockford USA). Images and quantification of bands were analyzed using ImageJ software.

#### RT-PCR Analysis

SN4741 cells at a density of 2.4 × 10^3^ cells/cm^2^ were seeded in 60 mm wells by triplicate during 2 weeks in normal conditions and treated with GO and derivatives using a 50 μg/ml concentration in the long term in culture (21 days). Cells incubated in normal conditions without graphene were used as a negative control. RNA extraction and RT-PCR to obtain the corresponding cDNA from 200 ng RNA were carried out using RNase Kit (Qiagen) and superscript Reverse Transcriptase (Invitrogen) and Oligo (dT) 16 (Promega, Madison, USA). Real-time quantitative polymerase chain reaction (RT-qPCR) was performed using an AB 7500 Real-Time PCR system (Applied Biosystems). The amplification was carried out as recommended by the manufacturers using 20 μL of the reaction mixture with 10 μL of SYRB Green I Universal Taq Mix (Roche Diagnostic, Switzerland), 0.25 μM of appropriate primers, and 2 μL of cDNA. The following primer sequences were used: *Pitx3:* (5′-TTCCCGTTCGCCTTCAACTCG-3′ and 5′-GAGCTGGGCGGTGAGAATACAGG-3′); *Limx1b*: (5′-GGCACGAGGAGTGTTTGCAGT-3′ and 5′-GTTTGCAGTACAGTTTCCGATCC-3′); *Limx1a*: (5′-TTGCACTCTCGCACA-3′ and 5′-GCCTGCTTGCCGAAT-3′); *Tuj1 (*β*-III-Tubulin)* (5′-CCATTCAGAGTAAGAACAGTAGTTACT-3′ and 5′-GGATGTCACACACCGCTACCTT-3′); Th: (5′-GCTGGAGGATGTGTCTCACTTCTT-3′ and 5′-CAGAAAATCACGGGCAGACAGTA-3′); *Dat*/Slc6a3: (5′-GCCATGTACCCCAGGAAGGA-3′ and 5′-CTACCGTCTTCGTGAGGCAT-3′); *Nr4a2/Nurr:* (5′-CAGCTCCGATTTCTTAACTCCAG-3′ and 5′-GGTGAGGTCCATGCTAAACTTGA-3′) and *Gadph* as housekeeping gene (5′-GAGAAACCTGCCAAGTATGATGA-3′ and 5′-AGACAACCTGGTCCTCAGTGTA-3′). Each RT-qPCR experiment was done by triplicate for each analyzed gene. Data were analyzed according to Pfaffl (2001).

### Neuronal Dysfunction: Protection From Rotenone

SN4741 cells were seeded at a concentration of 2.4 × 10^3^ cells/cm^2^ for 7 days for all the conditions. Then, the control and GOd treated cells were treated with 1.2 μM Rot in 0.1% DMSO for 24 h as previously described (Rodríguez-Losada et al., 2020). α-synuclein levels were measured by Western blot using a monoclonal α-synuclein antibody that identified a band of around 18 KDa and was a predicted to identify a band of 14.5 KDa (clone syn S211, Thermo-Fisher), polyclonal cFos antibody involved in identified with a band around 50 KDa and predicted band of 62 KDa (Cell Signaling). Signals were detected with ECL systems (Super Signal West Pico Thermo-Fisher, Rockford, USA). Images and quantification of bands were analyzed using Image Lab 4.0.1 and the Fiji/ImageJ software in three different experiments.

### Statistical Analysis

The results obtained were expressed as means ± SD. The same experiment was repeated at least three times, except for the cell cycle assay, which was repeated twice. Data were analyzed using GraphPad Prism data analysis software (GraphPad Software, San Diego, CA). For the comparison of statistical significance between two groups, Student's *t*-tests for paired and unpaired data were used. To multiple comparisons were used the One-way and Two-way ANOVA with Fisher's *post-hoc* test.

## Results

### Analysis of Chemical Composition and Structure

The structural morphology changes of GO-film, PRGO-film, and FRGO-film are shown in Figure 1A. It is clearly observed how the GO-film becomes corrugated as a consequence of the reduction process due to the escape of oxygen-containing groups. Both PRGO-film and FRGO-film show a more significant number of wrinkles of crumbled regions compared to the GO-film. Not evident changes have been noticed in the morphology of PRGO-film and FRGO-film. However, for the high magnification SEMS, the FRGO-film showed small clear dots (shorter than 10 nm of diameter) on the graphene sheets' surface. Since the reduction, the temperature of the FRGO-film is significantly higher than that of the PRGO-film (1,100 and 300°C, respectively), carbon particles or other impurities might have been formed during the reduction process. So, to evaluate the effectiveness of the reduction process, we analyzed the O 1s/C 1s ratio obtained from the XPS study (Pei and Cheng, 2012). The GO film's O/C decreased from 0.37 to 0.19 when it is reduced at 300°C in argon (PRGO-film).

However, when the reduction temperature is increased to 1,100°C, the presence of oxygen significantly decreased to only 0.04 O/C for the FRGO-film (Figure 1B), which shows the effectiveness of this reduction process, as expected. For this FRGO-film also, the presence of nitrogen, chlorine, and sulfur has been decreased. However, for all the films, both nitrogen and sulfur are below 1% atomic concentration (see Supplementary Figure 1, the table with statistical differences between films).

### Viability

**Figure 2:**
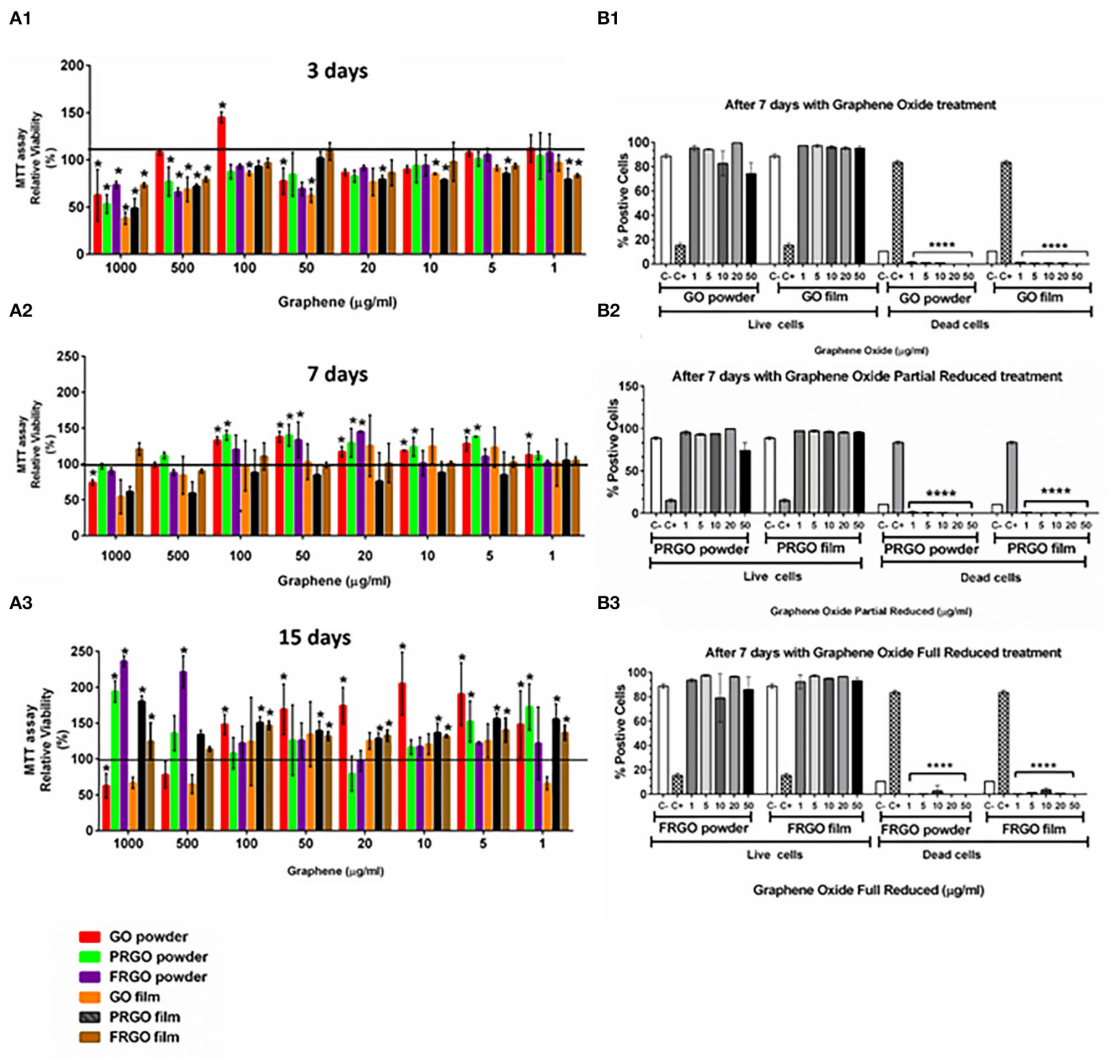
Effects of GOd treatment on cell viability of SN4741 cells. **(A)** MTT assay showing the relative cell viability (%) after 4 h **(A1)**, 7 days **(A2)**, and 15 days **(A3)**. Results are normalized to cells without treatment, represented as a black horizontal line. Data are expressed as mean ± SD, *n* = 3 experiments (each in triplicate determinations). *p < 0.05 compared to control using Two-way ANOVA. **(B)** Life/Dead assay using calcein-AM and ethidium homodimer-1 expressed as the percentage of a total number of stained cells counted by flow cytometry at day 7. Cells were cultured with GO **(B1)**, PRGO **(B2)**, and FRGO **(B3)** in powder or film forms. Data are expressed as means ± SD, *n* = 3. ****p < 0.0001, compared to control using Two-way ANOVA.

Viability was examined in broad dose-response experiments (1–1,000 ug/ml) performed at days 3, 7, and 15 using SN4741 cells seeded at a density of 5.3 × 10^3^ cells/cm^2^ (3 days) and 2.4 × 10^3^ cells/cm^2^ (for 7 and 15 days), to minimize cellular stress. At day 3, high substrate concentrations (500 and 1,000 μg/m) reduced cell viability by 40–60% (*p* < 0.05). However, GO-powder maintained viability at 500 μg/mL and even increased viability above control at 100 μg/mL (Figure 2A1). At lower concentrations (100–1 μg/mL), most of the conditions showed good viability (80–100%).

At 7 days in the culture, we observed a general increase in cell viability compared to control cultures without grapheme. Viability was particularly good (90–150%) for GO- and PRGO-powder at 100–5 μg/mL (Figure 2A2). At 15 days, we found that most substrates increased viability, but at different concentrations (Figure 2A3). A significant increase was detected at 1–100 μg/ml for GO-powder, PRGO-film, and FRGO-film treatments (*p* < 0.05). Surprisingly, PRGO-powder increased relative viability only at the highest (1,000 μg/ml) and the lowest concentrations (1 and 5 μg/ml).

To confirm these results, we performed additional viability measures with calcein-AM and examined cell death using ethidium homodimer 1, which binds to DNA only in cells that have lost the integrity of the plasma membrane. As a positive control, we used cells treated with 10% Triton X-100. Our results show precise maintenance of cell viability and the near-complete absence of cell death at day 7 in GO-powder or film (Figure 2B1), PRGO-powder or film (Figure 2B2) and FRGO-powder or film (Figure 2B3) in a range of concentrations from 50 to 1 μg/mL underlining the excellent biocompatibility of GO and GOd.

### Cell Proliferation

**Figure 3:**
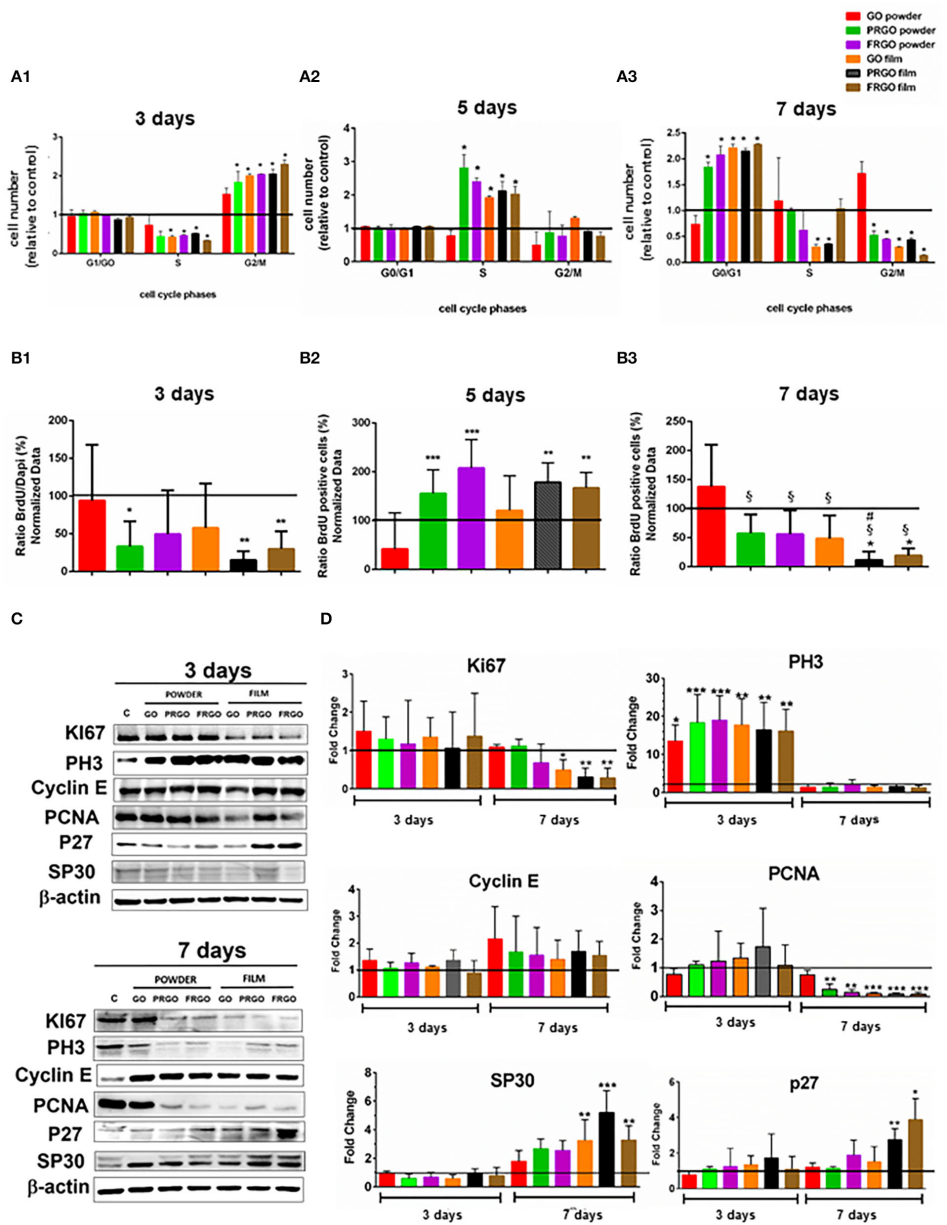
Effect of GOd treatment (50 μg/ml) on cell cycle distribution and proliferation of SN4741 cells **(A)**. Percentage of cells in G0/G1, S, and G2/M at day 3 **(A1)**; at day 5 **(A2)**, and day 7 **(A3)**. Data are expressed as mean ± SD, *n* = 2 experiments (by triplicate). Statistical analysis: *t*-Student, *p < 0.05 compared to control. **(B)** Percentage of BrdU positive cells at day 3 **(B1)**, 5 **(B2)**, and 7 **(B3)**. Data are expressed as mean ± SD, *n* = 3. *p < 0.05, **p < 0.01, ***p < 0.001 compared to control samples using ANOVA. **(C)** Representative Western blots of proteins involved in cell cycle regulation, including Ki67 (360 kDa), Cyclin E (48 kDa), PH3 Ser 10 (20 kDa), PCNA (36 kDa), P27 (27 kDa), and SP30 (30 kDa) at days 3 and 7. Data were normalized to β-actin (42 kDa). **(D)** Quantification of protein levels normalized to control and expressed as means ± SD, *n* = 3. *p < 0.05, **p < 0.01, ***p < 0.0001) compared to control. Statistical analysis was done with ANOVA for PH3, PCNA, P27, Cyclin E, and SP30 and Student *t*-test, for Ki67.

We first examined the cell cycle of propidium iodide-stained SN4741 cells by FACS; at day 3, analysis of the cell cycle (Figure 3A1) revealed that most SN4741 cells seeded with PRGO powder and film, FRGO powder and film and GO film (50 μg/mL) were in the G2/M phase (2.5-fold increase, ^*^p < 0.05) compared to the control. This increase was at the expense of cells in the S phase (0.5-fold decrease), while no change was detected in cells at G1/G0. These results suggest that SN4741 cells continue to proliferate on day 3, except for GO powder that does not vary with respect to the control. Except for GO powder, GOd induced a significant increase in DNA synthesis compared to control at day 5 (Figure 3A2). GO powder does not seem to alter the proliferative cycle, remaining similar to control cells.

In contrast, at day 7, most cells were in the G0/G1 phase (2.5-fold increase compared to control, ^*^p < 0.05), and a reduction in the number of cells in G2/M was detected (0.5-fold compared to the control condition, ^*^p < 0.05), suggesting that cells arrest at G0/G1. The exception to this was GO powder, which showed no significant difference compared to control in any phase. In addition, GO-film and PRGO-film showed a reduction in the number of cells in the S phase (Figure 3A3), suggesting a different cell cycle arrest role at G0/G1.

Cell proliferation was also examined by BrdU incorporation 4 h before fixation, after 3, 5, and 7 days of GOd treatment. At day 3 BrdU incorporation was significantly decreased (^*^p < 0.05; ^**^p < 0.01) compared to control (Figure 3B1). This was particularly pronounced in PRGO and FRGO powder and -film), which showed a significant increase at day 5 (Figure 3B2) and a subsequent decrease in BrdU incorporation at day 7 (Figure 3B3), suggesting a strong effect of PRGO (and FRGO) in decreasing proliferation.

Finally, to confirm these results, we also examined the levels of proteins involved in the cell cycle by Western blot (Figures 3C,D). Analysis of Ki67 protein, a proliferation marker degraded in G0, revealed no changes after treatment with GOd for 3 days, but a decrease after 7 days, especially with film forms (Figures 3C,D). We also examined PH3 (Ser10), a marker of mitosis, and found a significant increase after 3 days of treatment (up to 10–30-fold, ^**^p < 0.01), but a non-significant difference compared to control samples at day 7. Cyclin E, a protein required for progression through G1 and initiation of DNA replication (S phase), did not show any significant change. However, the replication marker, PCNA, was found downregulated at day 7 of treatment with all God, except for GO-powder. Finally, analysis of markers involved in senescence (SP30) or quiescence/differentiation (P27 cKip) revealed a significant raise (^*^p < 0.05) in cells treated with PRGO and FRGO films at day 7. Thus combined, our results indicate that PRGO- and FRGO-film induce an anti-proliferative effect on SN4741 cells at day 7 and suggest a possible pro-differentiation effect.

### Mitochondrial Metabolism and Bioenergetics

**Figure 4:**
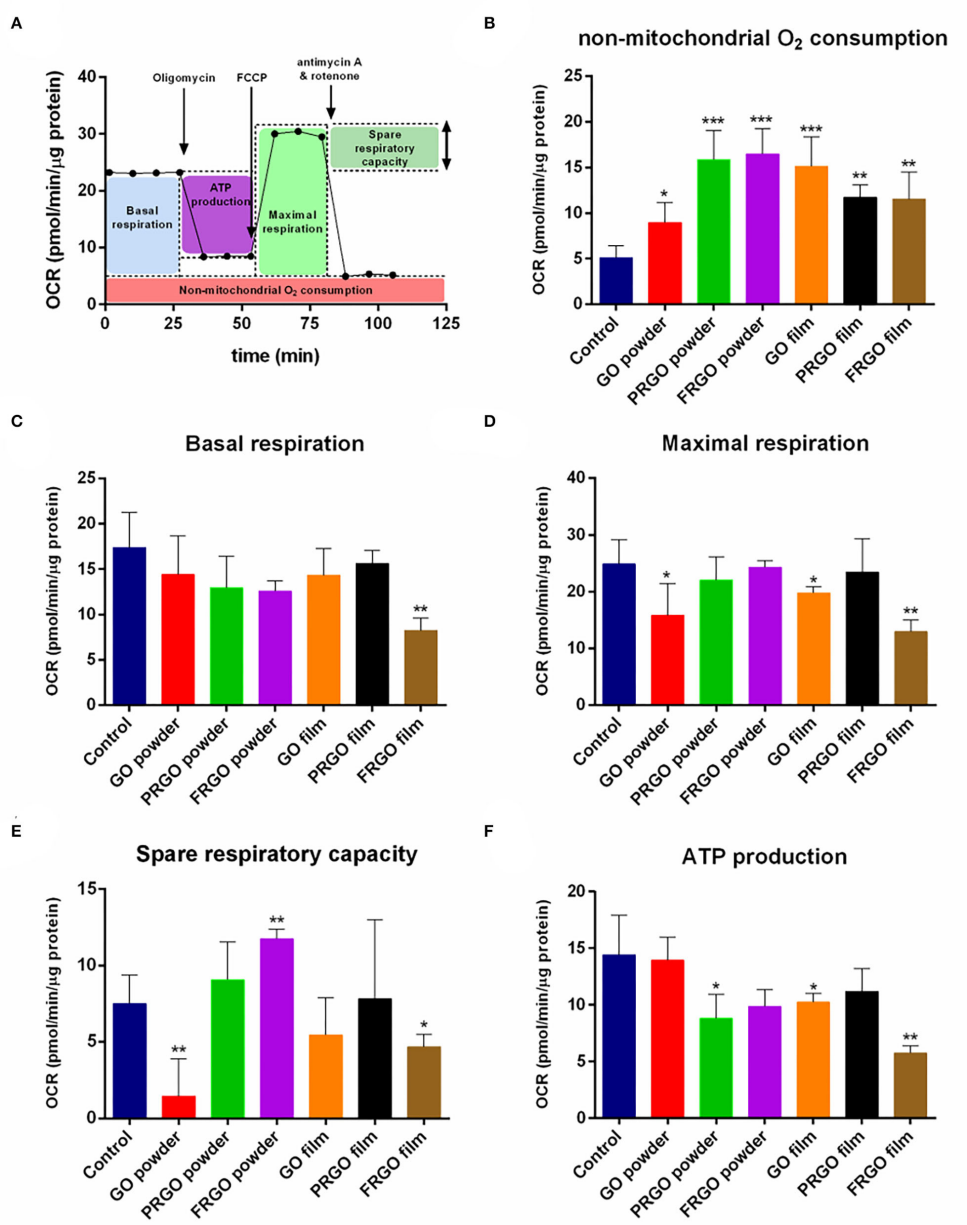
Effect of GOd treatment on the oxygen consumption rate (OCR) of SN4741 cells. **(A)** Representative scheme of metabolic parameters calculated from the OCR results. **(B)** Non-mitochondrial oxygen consumption, **(C)** basal respiration, **(D)** maximal respiration, **(E)** spare respiratory capacity, and **(F)** ATP production of SN4741 cells treated for 7 days with 50 μg/ml of GOd. Data are normalized to protein amount and expressed as means ± SD, *n* ≥ 3. *p < 0.05, **p < 0.01, ***p < 0.001 compared to control using ANOVA.

Mitochondrial respiration is essential for multiple cellular functions, including neuronal differentiation (Agostini et al., 2016), and previous studies have indicated that graphene can disrupt mitochondrial activity both in the zebrafish brain (Sun et al., 2019). We thus set out to examine whether GOd treatment impairs or allows for a correct energetic mitochondrial metabolism as assessed by the extracellular flux analyzer, Seahorse. In this experiment, different steps of the mitochondrial electron transport chain (ETC) are inhibited, and oxygen consumption rate (OCR) is measured in real-time (Supplementary Figure 2). Inhibitors used sequentially in this experiment include (i) Oligomycin, an inhibitor of the ATP synthase (complex V), which causes a decrease in OCR. (ii) FCCP, an uncoupling agent that collapses the proton gradient and disrupts the mitochondrial membrane potential, stimulating the ETC activity and thus increasing OCR. (iii) The combination of antimycin A, an inhibitor of complex III, and Rot, which inhibits complex I, shuts down mitochondrial respiration and drops OCR to the minimum level linked to mitochondrial respiration. This strategy allows calculating different metabolic parameters, such as non-mitochondrial oxygen consumption, basal and maximal respiration, spare respiratory capacity, and ATP production (Figure 4A).

Seahorse experiments showed a significant difference in the highest non-mitochondrial oxygen consumption after GOd treatments with the control condition (^*^p < 0.05) (Figure 4B). This non-mitochondrial oxygen consumption corresponds to an oxygen consumption non-linked to the ETC or mitochondrial respiration, such as substrate oxidation and peroxisomal and cell surface oxidation, which often correlates with a high reactive oxygen species (ROS) production (Funes et al., 2015). FRGO-film was the only GOd to decrease basal respiration, understood as the respiration rate under basal conditions, with a high significance (^**^p < 0.01) (Figure 4C). However, GO-powder and GO-film also decrease maximal respiration, i.e., the highest respiratory capacity of the cell (^*^p < 0.05) (Figure 4D). The other three GOd did not have a significant effect in either basal or maximal respiration (Figures 4C,D). Markedly, GO-powder and FRGO-film significantly diminished the spare respiratory capacity of these cells (^*^p < 0.05) (Figure 4E). This parameter indicates the capacity of a cell to respond to energetic demand. Usually, low maximal respiration correlates with a low spare respiratory capacity, as seen with these couple of GOd. Interestingly, FRGO-powder did not affect maximal respiration, but it increased the spare respiratory capacity of these cells (^**^p < 0.01) (Figure 4E).

Mitochondrial respiration is linked to ATP production mediated by the complex V of the ETC. PRGO-powder, GO-film, and FRGO-film significantly diminished the ATP linked respiration (^*^p < 0.05) (Figure 4F). This ATP linked respiration can be interpreted as the ATP production due to the activity of the ETC. It is important to note that PRGO-film did not reduce basal (Figure 4C) or maximal respiration (Figure 4D), spare respiratory capacity (Figure 4E), and ATP production (Figure 4F) indicating that PRGO-film is a fully biocompatible surface with no negative impact on mitochondrial function.

### Neuronal and Dopaminergic Differentiation

**Figure 5:**
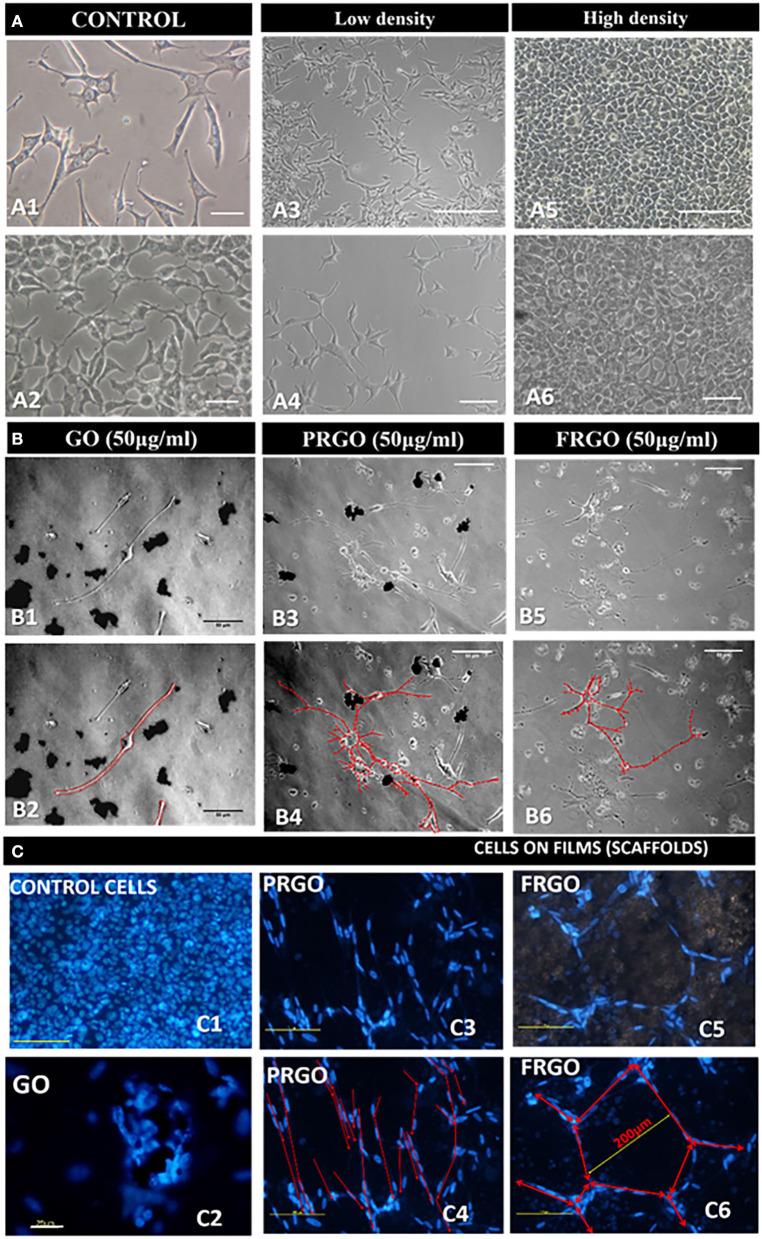
Effect of GOd treatment on the morphology and cytoarchitecture of SN4741 cells in culture. **(A)** Representative microphotographs of the morphology of SN4741 cells grown in plastic multiwell plates. **(A1,A2)** Control SN4741 cells cultured without GOd treatment in standard 10% FBS for 7 days. The different morphologies are highlighted at low **(A3,A4)** and high **(A5,A6)** density in culture in comparison with samples culture with GOd **(B)**. Scale bars: 25 μm in **(A1,A2)**, 250 μm in **(A3,A5)**, 50 μm in **(A4,A6)**. **(B)** Analysis of the contour of SN4741 cells treated with GO **(B1,B2)**, PRGO **(B3,B4)**, and FRGO **(B5,B6)** in powder and flakes forms (50 μg/ml) for 4 weeks. Please note that control cells (cultured without GOd) have limited survival capacity and die before 1 month. The contour of the neuronal processes is shown in red in **(B2,B4,B6)**. Scale bar in **(B1–B6)**, 50 μm. **(C)** Cytoarchitecture of SN4741 cells labeled with Hoechst 33342 in the control condition (untreated) or treated with GOd. **(C1)** Control cells are shown at 10 days since they do not survive cultivation for 6 weeks. **(C2–C6)** GOd microflakes-treated cells survive for up to 6 weeks in culture and organize as clusters on GO film **(C2)**; as lines on PRGO film **(C3,C4)** or as polygons on FRGO **(C4,C5)**. Cytoarchitecture is highlighted in red in **(C4,C6)**. Scale bar in **(C2)**, 75 μm; the rest of **(C)**, 100 μm.

Next examined the influence of GOd (50 μg/mL) on morphological changes in the cellular cytoarchitecture and the development of neuron-like morphologies in SN4741 cells grown on plastic wells for long periods of time (6 weeks). We found that while control ells SN4741 cells retain a fibroblast-like morphology (Figure 5A), GOd (micro flake) treatments induced the formation of neurite-like processes (Figure 5B); and the cytoarchitecture of cells treated with GO powder changed dramatically (Figure 5C).

**Figure 6:**
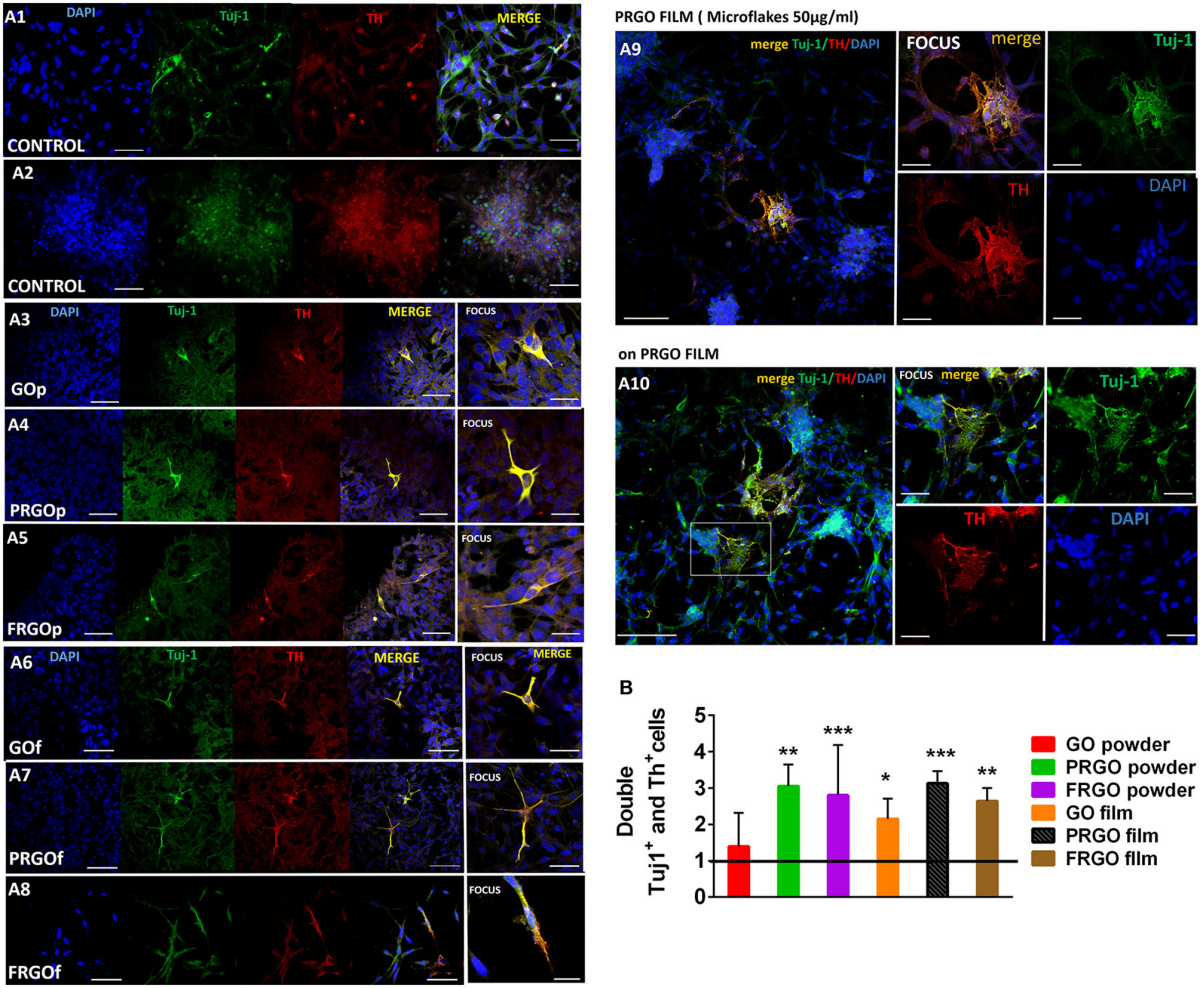
**(A)** Microscopy immunofluorescence images of SN4741 cells treated with GOd (50 μg/ml) where is shown the specificity of the triple labeling after the fix and cultured in cover glass. Cells are identified by nuclear DAPI staining (blue) and examined for co-staining (yellow) of Tuj-1 (green) and TH (red). **(A1)** Control cells with the low confluence in culture. The marker TH+ is localized into nuclei in most of case. Scale Bar 50 μm. **(A2)** Control cells in the long term in culture close to peeling off the plate with high confluence, therefore it is noticeable the small pre-apoptotic nuclei in the cells. Scale Bar 50 μm. Cells cultured on glass coverslips with GO powder **(A3)**, PRGO powder **(A4)**, FRGO powder **(A5)**, GO film **(A6)**, PRGO film **(A7)**, and FRGO film **(A8)**. Scale bar in **(A3–A8)**, 50 μm; and in the focus photography, 25 μm. **(A9)** Cells cultured in glass coverslips with PRGO film showing some neuronal processes. Scale bar, 100 μm; and scale in focus image, 50 μm. **(A10)** Cells seeded on PRGO film show morphologies similar to primary neurons. Scale bar, 250 μm; scale in focus photography, 50 μm. **(B)** Quantitative colocalization analysis of the number of TH+ and Tuj1+ cells acquired with an automatic threshold (Fiji-ImageJ, NIH). Results are expressed as Pearson's coefficient of colocalization (PCC). Data are normalized to control condition and expressed as mean ± SD, *n* = 6. *p < 0.05, **p < 0.01; ***p < 0.0001 compared to control, using Student *t*-test.

To analyze the degree of DA differentiation of SN4741 cells growing in GOd, we performed double immunostaining for the neuronal marker and βIII-tubulin (Tuj1) and the DA marker, TH (Figure 6A). Our results reveal a 2.5–4.5-fold increase in the colocalization of TH and Tuj1 by PRGO- and FRGO-powder and film (^**^p < 0.01; ^***^p < 0.0001), and by GO film compared to control (^*^p < 0.05) (Figure 6B, see also Supplementary Figure 3). Moreover, cell found to be double-positive for Tuj-1 and TH in PRGO- and FRGO-film scaffoldings exhibited neurite-like processes with branches, network formation, and connections (see Supplementary Figures 4, 5). However, the most notable examples of neural differentiation, with cell cluster formation, multiple cell contacts and extensive colocalization of TH and Tuj1, were only found in PRGO-film treated cells (Figures 6A9,A10, flakes panel).

**Figure 7:**
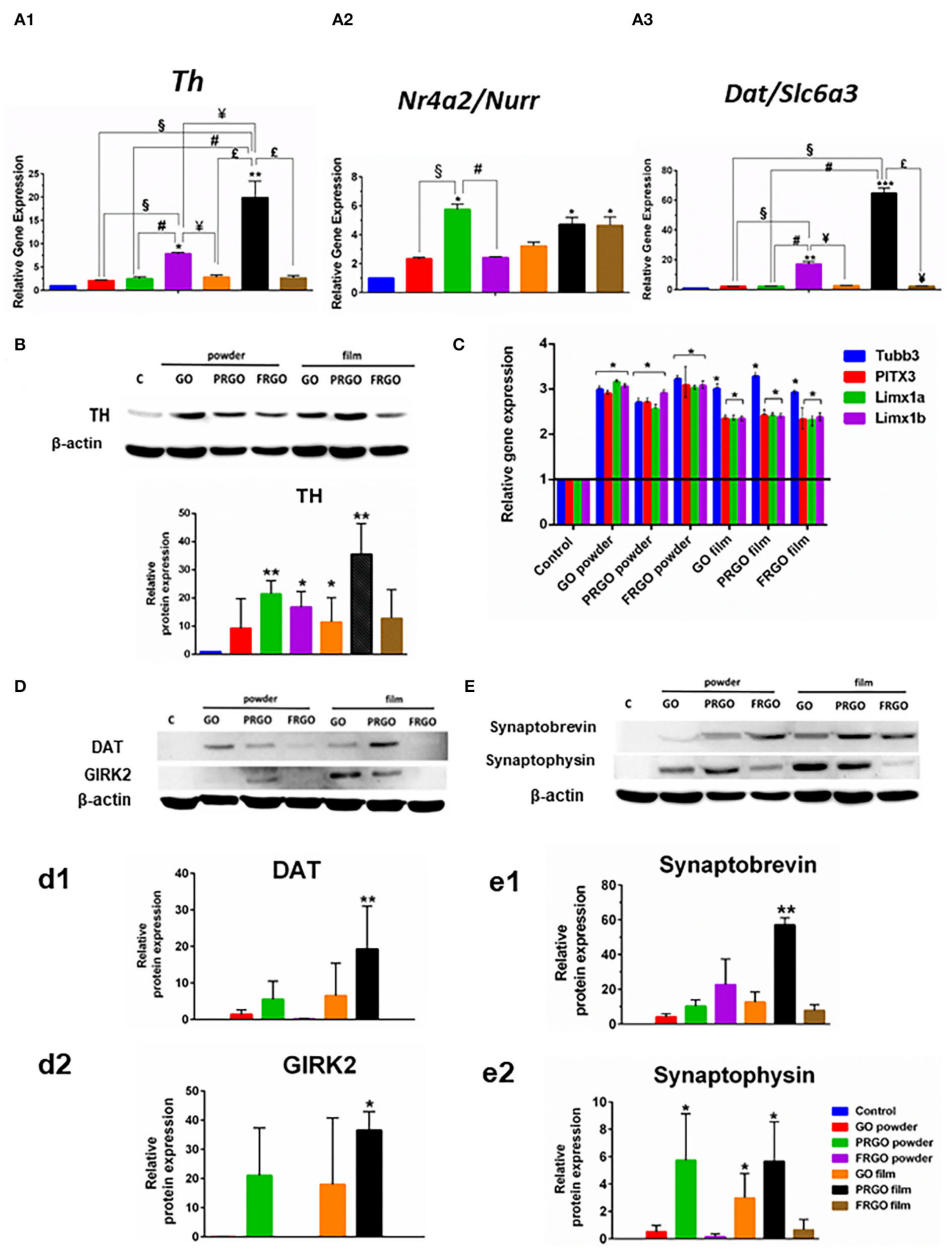
Effect of GOd treatment on the expression of genes and proteins involved in DA differentiation and maturation. **(A)** Relative expression of *Th* **(A1)**, *Nr4a2/Nurr1* **(A2)**, and *Dat*/Slc6a3 **(A3)** analyzed by Real-time RT-PCR in SN4741 treated or not with GOd (50 μg/ml) for 21 days. **(B)** Relative gene expression of *Tubb3, Pitx3, Lmx1a*, and *Lmx1b* analyzed by Real-time RT-PCR in SN4741 cells treated or not with GOd (50 μg/ml) for 15 days. **(C)** Representative Western blot and quantification of TH **(C)**, DAT **(D,d1)**, GIRK2 **(D,d2)**, synaptobrevin **(E,e1)**, and synaptophysin **(E,e2)** protein levels in SN4741 cells treated or not with GOd (50 μg/ml) for 21 days. Data are normalized to β-actin and expressed as means ± SD, *n* =3 **(A,B)**, *n* = 4 **(C)**, or *n* = 6 **(D)**. *p < 0.05, **p < 0.01, ***p < 0.001 for GOd vs. control; ^§^*p* < 0.001 for GO-powder vs. other GOd; ^#^*p* < 0.001 for PRGO-powder vs. other GOd; ^¥^*p* < 0.001 for FRGO-powder vs. other GOd; *^$^p* < 0.001 for PRGO-film vs. FRGO-film. The statistical test in all cases was Two-way ANOVA.

To determine whether TH positive cells acquired a midbrain DA phenotype (Figure 7A1), we examined whether the expression of markers that characterize this cell type was enhanced by exposure to GOd at a concentration of 50 μg/ml. Notably, the gene expression levels of *Nr4a2/Nurr1*, a transcription factor required from midbrain DA neuron development, increased 10-fold in PRGO-powder and film and FRGO-film (Figure 7A2). The dopamine transporter, *Dat/Slc6a3*, expressed in mature DA neurons, rose 60-fold (^****^p < 0.0001) with PRGO-film and 20-fold (^**^p < 0.01) with FRGO-powder compared to control cells (Figure 7A3). Moreover, all GOd conditions increased by 2.5–3-fold the expression of critical midbrain transcription factors, such as *Lmx1a* (Andersson et al., 2006) and *Lmx1b* (Smidt et al., 2000) and of the midbrain DA neuron-specific transcription factor *Pitx3* (Smidt et al., 2004) (Figure 7B). This data correlated with the morphological changes described above and with remarkable increases in the levels of TH protein (35-fold increase by PRGO film, Figure 7C) and DAT protein levels (20-fold increase by PRGO film, Figure 7D). The degree of neuronal maturation was further confirmed by the increase in the levels of the potassium channel protein typical of mDA neurons, GIRK2 (35-fold increase by PRGO film, Figure 7D), and of the synaptic proteins synaptobrevin (60-fold increase by PRGO film, Figure 7D) and synaptophysin (6-fold increase by PRGO film, Figure 7D). These results indicate that PRGO film is the best substrate for promoting DA differentiation of SN4741 cells.

### Protection From the Dopaminergic Neurotoxin Rotenone

**Figure 8:**
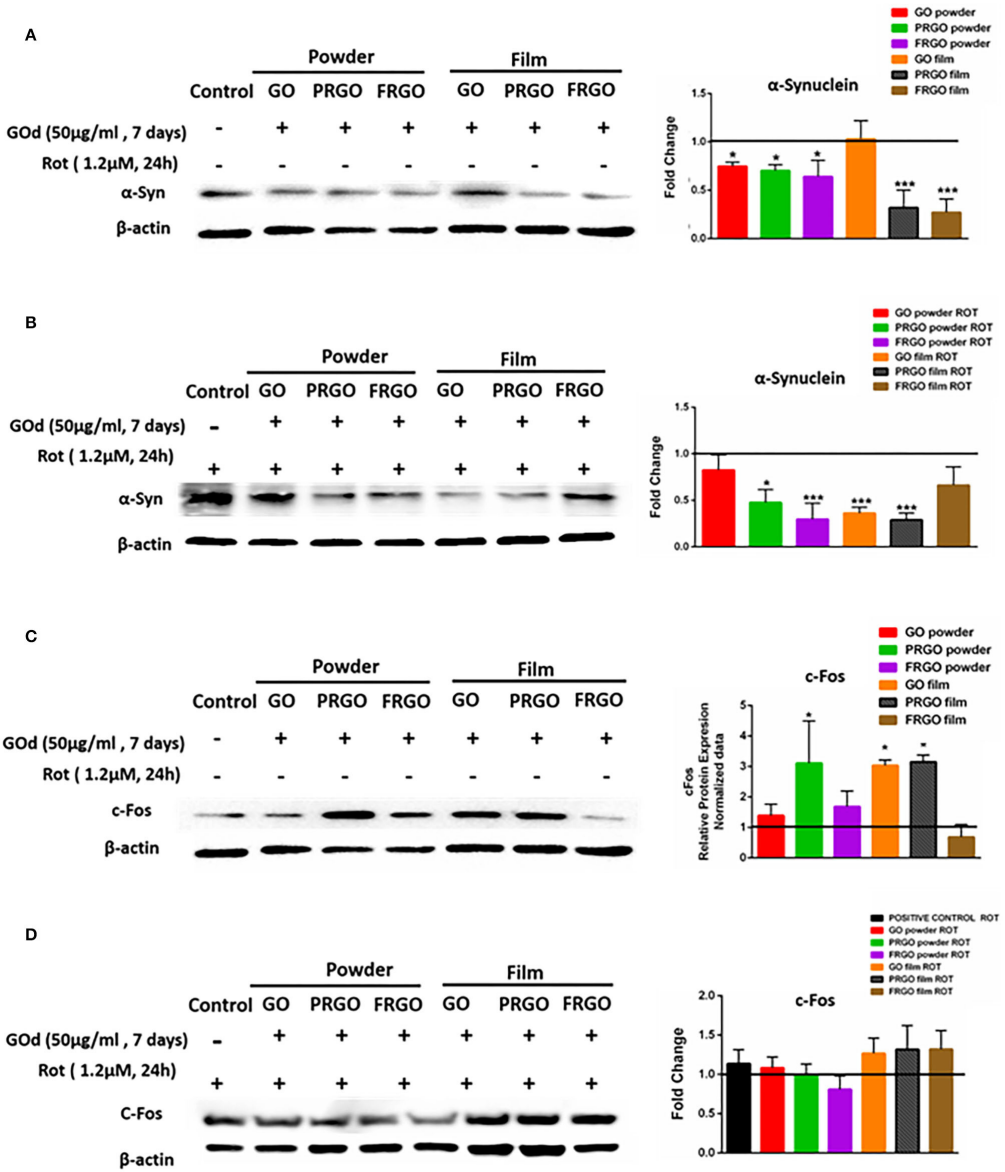
Analysis of α-Synuclein and c-Fos protein levels in SN4749 cultures in the absence or presence of GOd. **(A,B)** Representative western blots of α-Syn (18 KDa) and β-actin (43 KDa), showing untreated control and GOd treated (50 μg/ml) SN4741 cells at 7 days in the absence **(A)** or presence of Rot (1.2 μM) **(B)**. **(C,D)** Representative western blots of c-Fos (62 KDa) and β-actin (43 KDa), showing untreated control and GOd treated (50 μg/ml) SN4741 cells at 7 days in the absence **(C)** or presence of Rot (1.2 μM) **(D)**. Protein levels were normalized to the negative control (untreated), shown as a black line. Data represent the mean ± SD of three independent experiments per condition, *p < 0.5; ***p < 0.001 compared to control, using ANOVA.

We next investigated whether GOd could mitigate the effects of Rot, a DA neurotoxin, which causes mitochondrial dysfunction. SN4741 cells were found to produce endogenous α-Syn at 7 days in culture (Figure 8A). The basal production of α-Syn was reduced by all GOd, except for GO-film at a concentration of 50 μg/ml. Addition of the neurotoxin Rot to the culture medium increased the baseline levels of α-Syn in SN4741 cells, but its levels significantly decreased by GOd substrates, expect for FRGO film (Figure 8B). In addition, a reduction of α-Syn levels was also found in cells cultured on PRGO film and GO powder. cFos levels significantly increased in PRGO-powder and -film as well as in GO-film, but not in the rest of the samples, compared to untreated cells (Figure 8C). The baseline level of cFos was found to be higher in cultures exposed to Rot, but GOd had no effect of the levels of cFos, compared to cells cultured in the absence of GO derivatives (Figure 8D).

## Discussion

Our study compares three basic types of graphene (GO, PRGO, FRGO) in its film and powder state and examines its potential application as a scaffold to support the growth and differentiation of immature clonogenic dopaminergic cells SN4741, which presents a fibroblastic morphology and anecdotic expression of TH (Son et al., 1999), and can thus be used as a model of DA differentiation, as previously described (Schulte et al., 2005; Sousa et al., 2010; Salašová et al., 2017). We found that film micro-flakes provided the best substrate for cell survival and differentiation. The GOd film microflakes used in our study were of similar size to those previously described (Nair et al., 2015), and found to lack cell genotoxicity (Chang et al., 2011; Lalwani et al., 2015; Syama and Mohanan, 2016). So far, regenerative medicine studies have used GO (Sahni et al., 2013) and reduced GO without determining the degree of reduction (Ryu and Kim, 2013). In our study, the atomic composition and C/O ratio (hydrophobic and hydrophilic features, as well as the degree of GO reduction (full/partial), were considered.

At a functional level, we first focused on examining whether different GOd sustains the survival and affect the proliferation of DA cells. Our results indicate that concentrations bellow 100 μg/mL and longer times in culture (day 7 and 15) improve cell survival as assessed by MTT and calcein-AM assays, and decrease cell death, as examined by ethidium homodimer. Notably, parameters, such as BrdU incorporation, and Ki67, PH3, or PCNA protein levels decreased at day 7 *in vitro*, particularly by PRGO- and FRGO-film, compared to either untreated control in the day 3, indicating that both GOd treatment and time in culture also decrease proliferation. While results at early stages of cultivation are difficult to interpret, our results suggest that the increase in MTT and calcein-AM at day 7, when proliferation decreases, are predominately contributed by the survival of DA cells.

We also examined whether the identified decreased proliferation at day 7 is accompanied by an increase in the differentiation of SN4741 cells and their maturation into a neuronal midbrain DA phenotype. Dramatic changes in cell morphology were detected at day 7, when cells acquired neuronal morphology with processes, branches, and connections, as well as the expression of neuronal markers, such as Tuj1 and TH in these cells, as well as increased levels of mature DA neuron markers, such as DAT and GIRK2, and synaptic proteins, such as synaptophysin and synaptobrevin (Becher et al., 1999), particularly in cells treated with PRGO-film microflakes. In agreement with our results, previous have shown that GO promotes neurodifferentiation (Gordon et al., 2013; Feng et al., 2018), which allows the axonal sprouting (Li et al., 2011). Moreover, we found that GOd treatment induced the expression of typical midbrain transcription factors, such as *Lmx1a, Lmx1b, Nurr1*, and *Pitx3*, indicating that SN4741 cells effectively mature into neurons with a midbrain DA phenotype (Smidt et al., 2000, 2004; Arenas et al., 2015). It is worth noting that SN4741 cells are mouse *substantia nigra* neurons immortalized with the large T antigen (Son et al., 1999), which makes them proliferate. Indeed, in our experience, we have never observed such a degree of morphological and molecular differentiation and maturation of SN4741 cells. Thus, our results indicate that GOd are very powerful inducers of differentiation, being PRGO film more efficient than GO-film or FRGO-film. A plausible explanation for the differences in the materials is the atomic percentage and surface of structures, but also it could depend on the effect of functional groups (Serrano et al., 2014). The atomic study (SEM) shows that PRGO-film does not have chlorine and has the highest atomic levels of nitrogen and sulfur, compared to GO and FRGO.

Previous studies have shown that graphene can disrupt mitochondrial activity both in tumor cells (Lammel et al., 2013; Zhou et al., 2014) and in the zebrafish brain (Sun et al., 2019), both resulting in cytoskeletal abnormalities. Additionally, GO has been reported to trigger Parkinson's disease-like symptoms in zebrafish larvae, associated with the deregulation of metabolism (Ren et al., 2016). We, therefore, examined whether GOd impaired mitochondrial activity in a differential manner in SN4741 cells. We first found that all GOd increased non-mitochondrial oxygen consumption, with GO powder and PRGO- and FRGO-film showing small changes. These results suggest a possible increase in oxidative stress (Chang et al., 2011; Lammel et al., 2013), which we found is not sufficient to cause cell death.

Regarding oxygen consumption linked to mitochondrial respiration, GO-powder, and FRGO-film decreased mitochondrial respiration and the spare respiratory capacity. A low spare respiratory capacity, understood as the capacity of a cell to respond to an energetic demand, is usually found in some pathologies, including neuronal diseases (Desler et al., 2012). Therefore, our data suggest that GO-powder and FRGO-film are not suitable for DA neurons. In fact, from the six GOd used in this study, only PRGO, especially in the film form, did not alter mitochondrial metabolism, and FRGO-powder was the only one that increased the spare respiratory capacity of these cells. Thus, our analysis of mitochondrial respiration indicates that PRGO-film is the best GOd tested for DA neurons and the one that may find a potential application to prevent oxidative stress in PD. Indeed, our data confirm that most GOd diminished α-syn protein levels (Figure 8), a protein induced by oxidative stress and involved in PD (Menges et al., 2017; Musgrove et al., 2019). Data that has been corroborated recently in studies with neuroblastoma SH5Y5 whose clones express a-Syn treated with Rot (Rodríguez-Losada et al., 2020). In our study, we find that PRGO-film diminished α-synuclein levels in DA cells exposed to Rot, whereas FRGO-film did not. These results point to a greater oxidative capacity of FRGO-film and a greater neuroprotective capacity of PRGO-film. Other studies have found that GO derivatives similar to PRGO downregulate the expression of several mitochondrial complex genes (mainly I, III, IV, and V), whereas no effect of rGO (similar to our FRGO) or pristine graphene was found (Szmidt et al., 2019). In addition, PRGO has been found to decrease lipid peroxidation, whilst FRGO significantly increased lipid peroxidation and produced membrane damage (Li et al., 2018). All these data suggest that PRGO film may impact on several different mechanisms to reduce oxidative stress and promote neuronal survival and differentiation.

## Conclusion

In this study, we find that graphene in its oxidized (GO), partially reduced or (PRGO) or fully reduced (FRGO) forms, as well as in film or powder format, have distinct effects on DA cells. In general, films were found to reduce proliferation and promote neuronal differentiation more than powders; and lower concentrations supported survival better than higher concentrations. Our results suggest that the PRGO-film is the most suitable candidate to cultivate DA cells due to its capacity to promote the acquisition of a midbrain DA phenotype in SN4741 cells without inducing adverse effects on cell metabolism or mitochondrial function. Finally, we found a neuroprotective effect of PRGO-film on DA cells as shown by a reduction of the α-syn protein levels. We thus suggest that PRGO-film is a promising scaffold or material to develop constructs for DA cell replacement therapy for Parkinson's disease.

## Data Availability Statement

The original contributions presented in the study are included in the article/Supplementary Material, further inquiries can be directed to the corresponding author/s.

## Author Contributions

NR-L, EA, JN, MA, JA, PG-A, and RG contributed to the conception and design of the study. RW and RG performed the experiments in Figure 1, NR-L and JA in Figures 2, 4–7, MO and MM in Figure 3, and NR-L and AC in Figure 7. EA, JA, RW, RG, and MO wrote the manuscript with help from the rest of the authors. NR-L, EA, and JN coordinated the different aspects of the study. All authors contributed to manuscript revision, read, and approved the submitted version.

## Conflict of Interest

RW was employed by the company Abalonyx AS when the work was performed and the remaining authors declare that the research was conducted in the absence of any commercial or financial relationships that could be construed as a potential conflict of interest. Abalonyx claim no rights to any of the results.

## Supplementary Material

Supplementary Figure 1 Table showing the significant differences in the chemical composition of the films used in the study (Two-way ANOVA, ^****^*p* < 0.0001).

Supplementary Figure 2 Oxygen consumption rate (OCR measure in pmols/min/μg of protein) for GO powder **(A)**; PRGO powder **(B)**; FRGO powder **(C)**; GO film **(D)**; PRGO film **(E)**; FRGO film **(F)**.

Supplementary Figure 3 Photographs show immunofluorescence of cells stained with DAPI (nuclei marker in blue) Tuj-1/βIII tubulin 1 (green) and TH (red), showing the neuronal process in regions with a high number of positive cells after a long time in culture (4 weeks). **(A)** GO film (microflakes), scale bar 100 μm; **(B)** PRGO film (microflakes), scale bar 50 μm; **(C)** FRGO film (microflakes), scale bar 50 μm.

Supplementary Figure 4 Morphological changes of SN4741 cells when cultured on Film scaffolds. Photographs show immunostaining of cells stained with DAPI (nuclei in blue) Tuj-1/βIII tubulin 1 (green) and TH (red), showing neuronal processes. **(A)** Arborescent neurite-like structures in cells grown on PRGO-film. **(B)** Contact between TH-positive neurites from cells grown on FRGO film. **(C)** Cells cultivated on FRGO film.

Supplementary Figure 5 Comparison of fragments of 45 mm glass coverslip containing SN4741 cells cultured without (control) for 7 days or with PRGO for 4 weeks. **(A)** Cells were stained with Dapi (blue), Tuj-1 (green), and TH (red), and photographed at low magnification (scale bar, 1 mm). **(B)** A detail of part of the coverslip is shown at higher magnification (scale bar, 250 μm).
