# Supplementary material for: Graphene Oxide and Reduced Derivatives, as Powder or Film Scaffolds, Differentially Promote Dopaminergic Neuron Differentiation and Survival
Source: Front Neurosci. 2020 Dec 21;14:570409. doi: 10.3389/fnins.2020.570409 (PMC7779605; doi:10.3389/fnins.2020.570409)
Supplement: Supplementary file 1 [file Data_Sheet_1.pdf]

RAW DATA -STATISTICA ANALYSIS OF DATA

Figure 1

Two-way ANOVA	Ordinary				
Alpha	0,05				
Source of Variation	% of total variation	P value	P value summary	Significant?	
Interaction	5,008	< 0,0001	****	Yes	
Row Factor	94,62	< 0,0001	****	Yes	
Column Factor	0,3403	< 0,0001	****	Yes	
ANOVA table	SS	DF	MS	F (DFn, DFd)	P value
Interaction	1616	10	161,6	F (10, 18) = 296,4	P < 0,0001
Row Factor	30534	5	6107	F (5, 18) = 11198	P < 0,0001
Column Factor	109,8	2	54,91	F (2, 18) = 100,7	P < 0,0001
Residual	9,816	18	0,5453		

Number of families	1			
Number of comparisons per family	3			
**Alpha**	**0,05**			
**Tukey's multiple comparisons test**	**Mean Diff,**	**95% CI of diff,**	**Significant?**	**Summary**
**GO-film vs. PRGO-film**	**-0,3617**	**-1,131 to 0,4078**	**No**	**ns**
**GO-film vs. FRGO-film**	**-3,872**	**-4,642 to -3,103**	**Yes**	********
**PRGO-film vs. FRGO-film**	**-3,511**	**-4,280 to -2,741**	**Yes**	********

Test details	Mean 1	Mean 2	Mean Diff,	SE of diff,	N1	N2	q	DF
**GO-film vs. PRGO-film**	**16,79**	**17,15**	**-0,362**	**0,302**	**12**	**12**	**1,697**	**18**
**GO-film vs. FRGO-film**	**16,79**	**20,66**	**-3,872**	**0,302**	**12**	**12**	**18,17**	**18**
**PRGO-film vs. FRGO-film**	**17,15**	**20,66**	**-3,511**	**0,302**	**12**	**12**	**16,47**	**18**

Figure 2A1

Two-way ANOVA	Ordinary				
Alpha	0,05				
Source of Variation	% of total variation	P value	P value summary	Significant?	
Interaction	31,16	< 0,0001	****	Yes	
Row Factor	32,31	< 0,0001	****	Yes	
Column Factor	18,66	< 0,0001	****	Yes	
ANOVA table	SS	DF	MS	F (DFn, DFd)	P value
Interaction	19532	42	465	F (42, 112) = 4,653	P < 0,0001
Row Factor	20252	7	2893	F (7, 112) = 28,95	P < 0,0001
Column Factor	11696	6	1949	F (6, 112) = 19,51	P < 0,0001
Residual	11193	112	99,94		

CONTROL VS GO POWDER	Significant?	P value	Mean1	Mean2	Difference	SE of difference	t ratio	df
1000	*	< 0,0001	62,622	100	-37,378	7,1845	5,2026	32
500	ns	0,2532	108,36	100	8,3605	7,1845	1,1637	32
100	*	< 0,0001	145,07	100	45,067	7,1845	6,2729	32
50	*	0,0037	77,53	100	-22,47	7,1845	3,1276	32
20	ns	0,0744	86,748	100	-13,252	7,1845	1,8446	32
10	ns	0,1848	90,264	100	-9,7363	7,1845	1,3552	32
5	ns	0,2865	107,79	100	7,7868	7,1845	1,0838	32
1	ns	0,1021	112,09	100	12,092	7,1845	1,683	32
CONTROL VS PRGO POWDER	Significant?	P value	Mean1	Mean2	Difference	SE of difference	t ratio	df
1000	*	< 0,0001	100	53,234	46,766	9,0413	5,1725	32
500	*	0,0159	100	76,978	23,022	9,0413	2,5463	32
100	ns	0,1761	100	87,492	12,509	9,0413	1,3835	32
50	ns	0,0995	100	84,663	15,337	9,0413	1,6963	32
20	ns	0,0671	100	82,86	17,14	9,0413	1,8957	32
10	ns	0,4949	100	93,757	6,243	9,0413	0,6905	32
5	ns	0,9104	100	101,03	-1,0255	9,0413	0,1134	32
1	ns	0,6407	100	104,26	-4,2596	9,0413	0,4711	32
CONTROL VS FRGO POWDER	Significant?	P value	Mean1	Mean2	Difference	SE of difference	t ratio	df
**1000**	*	< 0,0001	73,529	100	-26,471	5,2759	5,0173	32
500	*	< 0,0001	66,036	100	-33,964	5,2759	6,4377	32
100	ns	0,2129	93,295	100	-6,705	5,2759	1,2709	32
50	*	< 0,0001	69,36	100	-30,64	5,2759	5,8076	32
20	ns	0,1195	91,56	100	-8,4404	5,2759	1,5998	32
10	ns	0,275	94,14	100	-5,8598	5,2759	1,1107	32
5	ns	0,2935	105,63	100	5,6344	5,2759	1,068	32
1	ns	0,1492	107,8	100	7,7981	5,2759	1,4781	32
CONTROL VS GO FILM								
1000	*	< 0,0001	38,247	100	-61,753	4,8747	12,668	32

500	*	< 0,0001	68,797	100	-31,204	4,8747	6,4011	32
100	*	0,0074	86,06	100	-13,94	4,8747	2,8596	32
50	*	< 0,0001	62,385	100	-37,616	4,8747	7,7165	32
20	*	< 0,0001	76,673	100	-23,327	4,8747	4,7852	32
10	*	0,0052	85,373	100	-14,627	4,8747	3,0006	32
5	ns	0,0918	91,526	100	-8,4742	4,8747	1,7384	32
1	ns	0,5325	96,924	100	-3,0764	4,8747	0,6311	32
CONTROL VS PRGO FILM	Significant?	P value	Mean1	Mean2	Difference	SE of difference	t ratio	df
1000	*	< 0,0001	48,738	100	-51,262	4,0626	12,618	32
500	*	< 0,0001	71,895	100	-28,105	4,0626	6,9179	32
100	ns	0,0921	92,946	100	-7,0543	4,0626	1,7364	32
50	ns	0,6326	101,96	100	1,9608	4,0626	0,4826	32
20	*	< 0,0001	79,333	100	-20,667	4,0626	5,0872	32
10	*	< 0,0001	78,882	100	-21,118	4,0626	5,1981	32
5	*	0,0013	85,711	100	-14,289	4,0626	3,5172	32
1	*	< 0,0001	79,322	100	-20,678	4,0626	5,0899	32
CONTROL VS FRGO FILM	Significant?	P value	Mean1	Mean2	Difference	SE of difference	t ratio	df
1000	*	< 0,0001	73,394	100	-26,606	5,6326	4,7235	32
500	*	0,0009	79,367	100	-20,633	5,6326	3,6632	32
100	ns	0,5874	96,912	100	-3,0877	5,6326	0,5482	32
50	ns	0,1229	108,93	100	8,925	5,6326	1,5845	32
20	ns	0,0221	86,455	100	-13,545	5,6326	2,4048	32
10	ns	0,7256	98,005	100	-1,9946	5,6326	0,3541	32
5	ns	0,2751	93,746	100	-6,2542	5,6326	1,1104	32
1	*	0,0061	83,457	100	-16,543	5,6326	2,9369	32

Figure 2A2

Two-way ANOVA	Ordinary				
Alpha	0,05				
Source of Variation	% of total variation	P value	P value summary	Significant?	
Interaction	22,99	0,0044	**	Yes	
Row Factor	18,16	< 0,0001	****	Yes	
Column Factor	26,39	< 0,0001	****	Yes	
ANOVA table	SS	DF	MS	F (DFn, DFd)	P value
Interaction	23294	42	554,6	F (42, 112) = 1,888	P = 0,0044
Row Factor	18398	7	2628	F (7, 112) = 8,947	P < 0,0001
Column Factor	26740	6	4457	F (6, 112) = 15,17	P < 0,0001
Residual	32902	112	293,8		

GO-POWDER vs control	Significant?	P value	Mean1	Mean2	Difference	SE of difference	t ratio	df
1000	*	< 0,0001	73,9874	100	-26,0126	4,76174	5,46284	32
500	ns	0,865436	99,1866	100	-0,813431	4,76174	0,170827	32
100	*	< 0,0001	132,786	100	32,7855	4,76174	6,88521	32
50	*	< 0,0001	137,782	100	37,7822	4,76174	7,93454	32
20	*	0,00108847	117,098	100	17,0982	4,76174	3,59076	32
10	*	0,000494683	118,459	100	18,4595	4,76174	3,87663	32
5	*	< 0,0001	128,071	100	28,071	4,76174	5,89513	32
1	*	0,0153062	112,201	100	12,2012	4,76174	2,56234	32
PRGO-POWDER vs control	Significant?	P value	Mean1	Mean2	Difference	SE of difference	t ratio	df
1000	ns	0,61889	96,8957	100	-3,10425	6,17997	0,502308	32
500	ns	0,0703365	111,57	100	11,5704	6,17997	1,87224	32
100	*	< 0,0001	139,99	100	39,9901	6,17997	6,47091	32
50	*	< 0,0001	139,824	100	39,8241	6,17997	6,44405	32
20	*	< 0,0001	129,283	100	29,2829	6,17997	4,73835	32
10	*	0,000518178	123,855	100	23,8546	6,17997	3,85998	32
5	*	< 0,0001	138,048	100	38,0478	6,17997	6,15663	32
1	ns	0,0665983	111,736	100	11,7364	6,17997	1,8991	32
FRGO-POWDER vs control	Significant?	P value	Mean1	Mean2	Difference	SE of difference	t ratio	df
1000	ns	0,205492	89,8074	100	-10,1926	7,88687	1,29234	32
500	ns	0,131691	87,7988	100	-12,2012	7,88687	1,54703	32
100	ns	0,0159611	120,07	100	20,0697	7,88687	2,5447	32
50	*	0,000181345	133,383	100	33,3831	7,88687	4,23275	32
20	*	< 0,0001	144,904	100	44,9037	7,88687	5,69347	32
10	ns	0,893689	101,062	100	1,0624	7,88687	0,134705	32
5	ns	0,19909	110,342	100	10,342	7,88687	1,31129	32
1	ns	0,823246	101,776	100	1,77622	7,88687	0,225212	32

GO-FILM vs control	Significant?	P value	Mean1	Mean2	Difference	SE of difference	t ratio	df
1000	ns	0,0138476	54,3825	100	-45,6175	17,5164	2,60428	32
500	ns	0,374182	84,2132	100	-15,7868	17,5164	0,901263	32
100	ns	0,883396	97,4104	100	-2,58964	17,5164	0,147841	32
50	ns	0,866362	102,971	100	2,97146	17,5164	0,169639	32
20	ns	0,157321	125,365	100	25,3652	17,5164	1,44809	32
10	ns	0,170927	124,535	100	24,5352	17,5164	1,4007	32
5	ns	0,194593	123,207	100	23,2072	17,5164	1,32489	32
1	ns	0,924341	101,677	100	1,67664	17,5164	0,0957183	32
PRGO-FILM vs control	Significant?	P value	Mean1	Mean2	Difference	SE of difference	t ratio	df
1000	ns	0,00764217	120,817	100	20,8168	7,31152	2,84712	32
500	ns	0,188371	90,1726	100	-9,82736	7,31152	1,34409	32
100	ns	0,157197	110,591	100	10,591	7,31152	1,44853	32
50	ns	0,834108	98,4562	100	-1,54384	7,31152	0,211152	32
20	ns	0,871177	101,195	100	1,19521	7,31152	0,163469	32
10	ns	0,878276	101,129	100	1,12879	7,31152	0,154385	32
5	ns	0,742441	102,424	100	2,42365	7,31152	0,331483	32
1	ns	0,516414	104,798	100	4,7975	7,31152	0,656156	32
FRGO-FILM vs control		Significant?	P value	Mean1	Mean2	Difference	SE of difference	t ratio
1000	ns	0,00764217	120,817	100	20,8168	7,31152	2,84712	32
500	ns	0,188371	90,1726	100	-9,82736	7,31152	1,34409	32
100	ns	0,157197	110,591	100	10,591	7,31152	1,44853	32
50	ns	0,834108	98,4562	100	-1,54384	7,31152	0,211152	32
20	ns	0,871177	101,195	100	1,19521	7,31152	0,163469	32
10	ns	0,878276	101,129	100	1,12879	7,31152	0,154385	32
5	ns	0,742441	102,424	100	2,42365	7,31152	0,331483	32
1	ns	0,516414	104,798	100	4,7975	7,31152	0,656156	32

Figure 2 A3

Two-way ANOVA	Ordinary				
Alpha	0,05				
Source of Variation	% of total variation	P value	P value summary	Significant?	
Interaction	57,24	< 0,0001	****	Yes	
Row Factor	2,227	0,0829	ns	No	
Column Factor	20,13	< 0,0001	****	Yes	
ANOVA table	SS	DF	MS	F (DFn, DFd)	P value
Interaction	161222	42	3839	F (42, 120) = 7,949	P < 0,0001
Row Factor	6272	7	896	F (7, 120) = 1,855	P = 0,0829
Column Factor	56703	6	9450	F (6, 120) = 19,57	P < 0,0001
Residual	57952	120	482,9		

Dunnett's multiple comparisons test	Mean Diff,	95% CI of diff,	Significant?	Summary
CON**TROL** vs. GO **powder**	-46,99	-62,44 to -31,53	Yes	****
CON**TROL** vs. PRGO p**owder**	-35,57	-51,02 to -20,12	Yes	****
CON**TROL** vs. FRGO p**owder**	-45,45	-60,90 to -29,99	Yes	****
CON**TROL** vs. GO f**ilm**	-3,467	-18,92 to 11,98	No	ns
CON**TROL** vs. PRG**O film**	-27,91	-43,37 to -12,46	Yes	****
CON**TROL** vs. FRG**O film**	-32,31	-47,76 to -16,86	Yes	****

GO POWDER vs Control	Significant?	P value	Mean1	Mean2	Difference	SE of difference	t ratio	df
1000	*	0,0237642	62,6245	100	-37,3755	15,9008	2,35055	40
500	ns	0,173087	77,9438	100	-22,0562	15,9008	1,38712	40
100	*	0,00429049	148,155	100	48,1547	15,9008	3,02845	40
50	*	< 0,0001	169,186	100	69,1857	15,9008	4,35109	40
20	*	< 0,0001	174,282	100	74,2823	15,9008	4,67162	40
10	*	< 0,0001	205,067	100	105,067	15,9008	6,60769	40
5	*	< 0,0001	190,422	100	90,4218	15,9008	5,68663	40
1	*	0,00424827	148,213	100	48,2132	15,9008	3,03213	40
PRGO-POWDER vs Control	Significant?	P value	Mean1	Mean2	Difference	SE of difference	t ratio	df
1000	*	< 0,0001	194,113	100	94,1125	13,5355	6,95301	40
500	ns	0,0113839	135,911	100	35,911	13,5355	2,65309	40
100	ns	0,556558	108,026	100	8,02576	13,5355	0,592941	40
50	ns	0,0615139	126,04	100	26,0398	13,5355	1,92382	40
20	ns	0,13823	79,5255	100	-20,4745	13,5355	1,51265	40
10	ns	0,233499	116,374	100	16,3738	13,5355	1,20969	40
5	*	0,000411488	152,168	100	52,1676	13,5355	3,85413	40
1	*	< 0,0001	172,408	100	72,4078	13,5355	5,34947	40
FRGO-POWDER vs Control	Significant?	P value	Mean1	Mean2	Difference	SE of difference	t ratio	df
1000	*	< 0,0001	236,116	100	136,116	11,6579	11,6759	40
500	*	< 0,0001	221,127	100	121,127	11,6579	10,3901	40
100	ns	0,0660962	122,027	100	22,0269	11,6579	1,88944	40
50	*	0,0315232	125,981	100	25,9812	11,6579	2,22864	40
20	ns	0,816434	97,2759	100	-2,72409	11,6579	0,233669	40
10	ns	0,148749	117,165	100	17,1646	11,6579	1,47236	40
5	ns	0,0640516	122,203	100	22,2027	11,6579	1,90452	40
1	ns	0,0703524	121,675	100	21,6755	11,6579	1,85929	40
GO-FILM vs Control	Significant?	P value	Mean1	Mean2	Difference	SE of difference	t ratio	df
1000	*	0,0257212	66,8131	100	-33,1869	14,3247	2,31676	40
500	*	0,0192389	65,0557	100	-34,9443	14,3247	2,43945	40
100	ns	0,0982125	124,253	100	24,2531	14,3247	1,6931	40
50	ns	0,209061	134,446	100	34,4464	14,3247	2,40469	40
20	ns	0,0863067	125,19	100	25,1904	14,3247	1,75853	40
10	ns	0,158354	120,592	100	20,5917	14,3247	1,4375	40
5	ns	0,084214	125,366	100	25,3662	14,3247	1,7708	40
1	*	0,0225935	66,0223	100	-33,9777	14,3247	2,37197	40
PRGO-FILM vs Control	Significant?	P value	Mean1	Mean2	Difference	SE of difference	t ratio	df
1000	*	0,021178	113,884	100	13,884	5,78678	2,39926	40
500	ns	0,15271	108,436	100	8,43582	5,78678	1,45777	40
100	*	< 0,0001	142,326	100	42,3257	5,78678	7,31421	40
50	*	< 0,0001	144,757	100	44,7569	5,78678	7,73433	40
20	*	< 0,0001	135,969	100	35,9695	5,78678	6,2158	40
10	*	< 0,0001	132,513	100	32,5132	5,78678	5,61852	40
5	*	0,000301452	122,906	100	22,9057	5,78678	3,95828	40
1	*	0,000367077	122,525	100	22,5249	5,78678	3,89247	40
FRGO-FILM vs Control	Significant?	P value	Mean1	Mean2	Difference	SE of difference	t ratio	df
1000	*	0,000145362	124,663	100	24,6631	5,87368	4,19893	40
500	*	0,0238612	113,796	100	13,7962	5,87368	2,34881	40
100	*	< 0,0001	146,837	100	46,8365	5,87368	7,97397	40
50	*	< 0,0001	131,927	100	31,9273	5,87368	5,43566	40
20	*	< 0,0001	132,601	100	32,6011	5,87368	5,55037	40
10	*	< 0,0001	131,781	100	31,7809	5,87368	5,41073	40
5	*	< 0,0001	140,305	100	40,3046	5,87368	6,86191	40
1	*	< 0,0001	136,585	100	36,5847	5,87368	6,22858	40

Figure 2 B1

Source of Variation	% of total variation	P value	P value summary	Significant?
Interaction	40,61	< 0,0001	****	Yes
Row Factor	58,78	< 0,0001	****	Yes
Column Factor	0,3287	< 0,0001	****	Yes

ANOVA table	SS	DF	MS	F (DFn, DFd)	P value
Interaction	65597	18	3644	F (18, 56) = 458,8	P < 0,0001
Row Factor	94949	3	31650	F (3, 56) = 3985	P < 0,0001
Column Factor	530,9	6	88,49	F (6, 56) = 11,14	P < 0,0001
Residual	444,8	56	7,943		

Tukey's multiple comparisons test	Mean Diff,	95% CI of diff,	Significant?	Summary
GO Powder vs. GO Film	-4,993	-7,296 to -2,690	Yes	****
GO Powder vs. GO Powder DEAD	64,68	62,38 to 66,98	Yes	****
GO Powder vs. GO Film DEAD	64,62	62,32 to 66,93	Yes	****
GO Film vs. GO Powder DEAD	69,67	67,37 to 71,98	Yes	****
GO Film vs. GO Film DEAD	69,62	67,31 to 71,92	Yes	****
GO Powder DEAD vs. GO Film DEAD	-0,05667	-2,360 to 2,246	No	ns

Figure 2B2

Table Analyzed	Data 1				
Two-way ANOVA	Ordinary				
Alpha	0,05				
Source of Variation	% of total variation	P value	P value summary	Significant?	
Interaction	40,42	< 0,0001	****	Yes	
Row Factor	59,16	< 0,0001	****	Yes	
Column Factor	0,2724	< 0,0001	****	Yes	
ANOVA table	SS	DF	MS	F (DFn, DFd)	P value
Interaction	66203	18	3678	F (18, 56) = 860,2	P < 0,0001
Row Factor	96881	3	32294	F (3, 56) = 7553	P < 0,0001
Column Factor	446,1	6	74,35	F (6, 56) = 17,39	P < 0,0001
Residual	239,4	56	4,275		

Number of families	1			
Number of comparisons per family	6			
Alpha	0,05			
Tukey's multiple comparisons test	Mean Diff,	95% CI of diff,	Significant?	Summary
PRGO Powder vs. PRGO Film	-3,539	-5,228 to -1,849	Yes	****
PRGO Powder vs. PRGO Powder DEAD	66,13	64,45 to 67,82	Yes	****
PRGO Powder vs. PRGO Film DEAD	66,08	64,39 to 67,77	Yes	****
PRGO Film vs. PRGO Powder DEAD	69,67	67,98 to 71,36	Yes	****
PRGO Film vs. PRGO Film DEAD	69,62	67,93 to 71,31	Yes	****
PRGO Powder DEAD vs. PRGO Film DEAD	-0,05667	-1,746 to 1,633	No	ns

Test details	Mean 1	Mean 2	Mean Diff,	SE of diff,	N1	N2	q	DF
PRGO Powder vs. PRGO Film	80,1	83,64	-3,539	0,6381	21	21	7,842	56
PRGO Powder vs. PRGO Powder DEAD	80,1	13,97	66,13	0,6381	21	21	146,6	56
PRGO Powder vs. PRGO Film DEAD	80,1	14,02	66,08	0,6381	21	21	146,4	56
PRGO Film vs. PRGO Powder DEAD	83,64	13,97	69,67	0,6381	21	21	154,4	56
PRGO Film vs. PRGO Film DEAD	83,64	14,02	69,62	0,6381	21	21	154,3	56
PRGO Powder DEAD vs. PRGO Film DEAD	13,97	14,02	-0,05667	0,6381	21	21	0,1256	56

Figure 2B3

Tukey's multiple comparisons test	Mean Diff,	95% CI of diff,	Significant?	Summary
FRGO Powder LIFE vs. FRGO Film LIFE	-3,077	-6,857 to 0,7025	No	ns
FRGO Powder LIFE vs. FRGO Powder DEAD	65,61	61,83 to 69,39	Yes	****
FRGO Powder LIFE vs. FRGO Film DEAD	65,25	61,48 to 69,03	Yes	****
FRGO Film LIFE vs. FRGO Powder DEAD	68,69	64,91 to 72,47	Yes	****
FRGO Film LIFE vs. FRGO Film DEAD	68,33	64,55 to 72,11	Yes	****
FRGO Powder DEAD vs. FRGO Film DEAD	-0,3586	-4,138 to 3,421	No	ns

Test details	Mean 1	Mean 2	Mean Diff,	SE of diff,	N1	N2	q	DF
FRGO Powder LIFE vs. FRGO Film LIFE	79,58	82,66	-3,077	1,427	21	21	3,049	56
FRGO Powder LIFE vs. FRGO Powder DEAD	79,58	13,97	65,61	1,427	21	21	65,01	56
FRGO Powder LIFE vs. FRGO Film DEAD	79,58	14,33	65,25	1,427	21	21	64,65	56
FRGO Film LIFE vs. FRGO Powder DEAD	82,66	13,97	68,69	1,427	21	21	68,05	56
FRGO Film LIFE vs. FRGO Film DEAD	82,66	14,33	68,33	1,427	21	21	67,7	56
FRGO Powder DEAD vs. FRGO Film DEAD	13,97	14,33	-0,3586	1,427	21	21	0,3553	56

FIGURE 3 A1

GO-POWDER	Significant?	P value	Mean1	Mean2	Difference	SE of difference	t ratio	df
G1/GO	ns	0,783941	1	0,959437	0,0405626	0,141451	0,28676	6
S	ns	0,111521	1	0,736259	0,263741	0,141451	1,86453	6
G2/M	*	0,00922392	1	1,53412	-0,534115	0,141451	3,77596	6
PRGO-POWDER	Significant?	P value	Mean1	Mean2	Difference	SE of difference	t ratio	df
G1/GO	ns	0,758108	1	1,04147	-0,0414726	0,128649	0,322371	6
S	*	0,00505536	1	0,445943	0,554057	0,128649	4,30675	6
G2/M	*	0,000623199	1	1,83822	-0,83822	0,128649	6,51558	6
FRGO-POWDER	Significant?	P value	Mean1	Mean2	Difference	SE of difference	t ratio	df
G1/GO	ns	0,057347	1	1,07232	-0,0723245	0,0278313	2,59867	6
S	*	< 0,0001	1	0,428025	0,571975	0,0278313	20,5515	6
G2/M	*	< 0,0001	1	2,0088	-1,0088	0,0278313	36,2468	6
GO-FILM	Significant?	P value	Mean1	Mean2	Difference	SE of difference	t ratio	df
G1/GO	ns	0,5186	1	0,99383	0,0061703	0,00900068	0,685538	6
S	*	< 0,0001	1	0,471512	0,528488	0,00900068	58,7165	6
G2/M	*	< 0,0001	1	2,04797	-1,04797	0,00900068	116,433	6
PRGO-FILM	Significant?	P value	Mean1	Mean2	Difference	SE of difference	t ratio	df
G1/GO	ns	0,0571791	1	0,877504	0,122496	0,0501156	2,44428	6
S	*	< 0,0001	1	0,520636	0,479364	0,0501156	9,56517	6
G2/M	*	< 0,0001	1	2,0557	-1,0557	0,0501156	21,0654	6
FRGO-FILM	Significant?	P value	Mean1	Mean2	Difference	SE of difference	t ratio	df
G1/GO	ns	0,239956	1	0,941938	0,058062	0,0445188	1,30421	6
S	*	< 0,0001	1	0,340246	0,659754	0,0445188	14,8197	6
G2/M	*	< 0,0001	1	2,3049	-1,3049	0,0445188	29,3113	6

Number of comparisons per family	3
Alpha	0,05

Tukey's multiple comparisons test	Mean Diff,	95% CI of diff,	Significant?	Summary
Con**trol**				
G1/GO vs. S	0	-0,2758 to 0,2758	No	ns
G1/GO vs. G2/M	0	-0,2758 to 0,2758	No	ns
S vs. G2/M	0	-0,2758 to 0,2758	No	ns
GO **powder**				
G1/GO vs. S	0,2232	-0,05263 to 0,4990	No	ns
G1/GO vs. G2/M	-0,5747	-0,8505 to -0,2989	Yes	****
S vs. G2/M	-0,7979	-1,074 to -0,5220	Yes	****
PRGO p**owder**				
G1/GO vs. S	0,5955	0,3197 to 0,8713	Yes	****
G1/GO vs. G2/M	-0,7967	-1,073 to -0,5209	Yes	****
S vs. G2/M	-1,392	-1,668 to -1,116	Yes	****
FRGO p**owder**				
G1/GO vs. S	0,6443	0,3685 to 0,9201	Yes	****
G1/GO vs. G2/M	-0,9365	-1,212 to -0,6607	Yes	****
S vs. G2/M	-1,581	-1,857 to -1,305	Yes	****
GO f**ilm**				
G1/GO vs. S	0,5223	0,2465 to 0,7981	Yes	***
G1/GO vs. G2/M	-1,054	-1,330 to -0,7783	Yes	****
S vs. G2/M	-1,576	-1,852 to -1,301	Yes	****

PRGO film				
G1/GO vs. S	0,3569	0,08106 to 0,6327	Yes	**
G1/GO vs. G2/M	-1,178	-1,454 to -0,9024	Yes	****
S vs. G2/M	-1,535	-1,811 to -1,259	Yes	****
FRGO film				
G1/GO vs. S	0,6017	0,3259 to 0,8775	Yes	****
G1/GO vs. G2/M	-1,363	-1,639 to -1,087	Yes	****
S vs. G2/M	-1,965	-2,240 to -1,689	Yes	****

Test details	Mean 1	Mean 2	Mean Diff,	SE of diff,	q	DF
Control						
G1/GO vs. S	1	1	0	0,1094	0	21
G1/GO vs. G2/M	1	1	0	0,1094	0	21
S vs. G2/M	1	1	0	0,1094	0	21
GO powder						
G1/GO vs. S	0,9594	0,7363	0,2232	0,1094	2,884	21
G1/GO vs. G2/M	0,9594	1,534	-0,5747	0,1094	7,427	21
S vs. G2/M	0,7363	1,534	-0,7979	0,1094	10,31	21
PRGO powder						
G1/GO vs. S	1,041	0,4459	0,5955	0,1094	7,697	21
G1/GO vs. G2/M	1,041	1,838	-0,7967	0,1094	10,3	21
S vs. G2/M	0,4459	1,838	-1,392	0,1094	17,99	21
FRGO powder						
G1/GO vs. S	1,072	0,428	0,6443	0,1094	8,327	21
G1/GO vs. G2/M	1,072	2,009	-0,9365	0,1094	12,1	21
S vs. G2/M	0,428	2,009	-1,581	0,1094	20,43	21
GO film						
G1/GO vs. S	0,9938	0,4715	0,5223	0,1094	6,75	21
G1/GO vs. G2/M	0,9938	2,048	-1,054	0,1094	13,62	21
S vs. G2/M	0,4715	2,048	-1,576	0,1094	20,37	21
PRGO film						
G1/GO vs. S	0,8775	0,5206	0,3569	0,1094	4,612	21
G1/GO vs. G2/M	0,8775	2,056	-1,178	0,1094	15,23	21
S vs. G2/M	0,5206	2,056	-1,535	0,1094	19,84	21
FRGO film						
G1/GO vs. S	0,9419	0,3402	0,6017	0,1094	7,776	21
G1/GO vs. G2/M	0,9419	2,305	-1,363	0,1094	17,62	21
S vs. G2/M	0,3402	2,305	-1,965	0,1094	25,39	21

FIGURE 3 A2

GO POWDER	Significant ?	P value	Mean 1	Mean2	Difference	SE of difference	t ratio	df
G0/G1	ns	0,497908	1	0,742857	0,257143	0,356544	0,721209	6
S	ns	0,608448	1	1,19264	-0,192639	0,356544	0,540296	6
G2/M	ns	0,0902612	1	1,71919	-0,719188	0,356544	2,01711	6
PRGO-POWDER	Significant ?	P value	Mean 1	Mean2	Difference	SE of difference	t ratio	df
G0/G1	*	< 0,0001	1	1,84707	-0,847067	0,0507916	16,6773	6
S	ns	0,735014	1	1,01801	-0,0180111	0,0507916	0,354607	6
G2/M	*	< 0,0001	1	0,528571	0,471429	0,0507916	9,28162	6
FRGO-POWDER	Significant ?	P value	Mean 1	Mean2	Difference	SE of difference	t ratio	df
G0/G1	*	0,000721612	1	2,07992	-1,07992	0,170365	6,33884	6
G2/M	*	0,0177813	1	0,448739	0,55126	0,170365	3,23576	6
GO-FILM	Significant ?	P value	Mean 1	Mean2	Difference	SE of difference	t ratio	df
G0/G1	*	< 0,0001	1	2,21808	-1,21808	0,0310136	39,2758	6
S	*	< 0,0001	1	0,304229	0,695771	0,0310136	22,4344	6
G2/M	*	< 0,0001	1	0,305602	0,694398	0,0310136	22,3901	6
PRGO-FILM	Significant ?	P value	Mean 1	Mean2	Difference	SE of difference	t ratio	df
G0/G1	*	< 0,0001	1	2,2853	-1,2853	0,0742569	17,3088	6
S	ns	0,543829	1	1,04777	-0,0477681	0,0742569	0,643281	6
G2/M	*	< 0,0001	1	0,143137	0,856863	0,0742569	11,5392	6

Within each column, compare rows (simple effects within columns)				
Number of families	7			
Number of comparisons per family	3			
Alpha	0,05			
Tukey's multiple comparisons test	Mean Diff,	95% CI of diff,	Significant?	Summary
Control				
G0/G1 vs. S	0	-0,5485 to 0,5485	No	ns
G0/G1 vs. G2/M	0	-0,5485 to 0,5485	No	ns
S vs. G2/M	0	-0,5485 to 0,5485	No	ns
GO powder				
G0/G1 vs. S	-0,4498	-0,9983 to 0,09870	No	ns
G0/G1 vs. G2/M	-0,9763	-1,525 to -0,4278	Yes	***
S vs. G2/M	-0,5265	-1,075 to 0,02193	No	ns
PRGO powder				
G0/G1 vs. S	0,8291	0,2806 to 1,378	Yes	**

G0/G1 vs. G2/M	1,318	0,7700 to 1,867	Yes	****
S vs. G2/M	0,4894	-0,05904 to 1,038	No	ns
FRGO p**owder**				
G0/G1 vs. S	1,455	0,9061 to 2,003	Yes	****
G0/G1 vs. G2/M	1,631	1,083 to 2,180	Yes	****
S vs. G2/M	0,1766	-0,3719 to 0,7250	No	ns
GO f**ilm**				
G0/G1 vs. S	1,914	1,365 to 2,462	Yes	****
G0/G1 vs. G2/M	1,912	1,364 to 2,461	Yes	****
S vs. G2/M	-0,001374	-0,5499 to 0,5471	No	ns
PRG**O film**				
G0/G1 vs. S	1,792	1,244 to 2,341	Yes	****
G0/G1 vs. G2/M	1,708	1,159 to 2,256	Yes	****
S vs. G2/M	-0,08474	-0,6332 to 0,4637	No	ns
FRG**O film**				
G0/G1 vs. S	1,238	0,6890 to 1,786	Yes	****
G0/G1 vs. G2/M	2,142	1,594 to 2,691	Yes	****
S vs. G2/M	0,9046	0,3561 to 1,453	Yes	**

Test details	Mean 1	Mean 2	Mean Diff,	SE of diff,	q	DF
**Control**						
G0/G1 vs. S	1	1	0	0,2176	0	21
G0/G1 vs. G2/M	1	1	0	0,2176	0	21
S vs. G2/M	1	1	0	0,2176	0	21
**GO powder**						
G0/G1 vs. S	0,7429	1,193	-0,4498	0,2176	2,923	21
G0/G1 vs. G2/M	0,7429	1,719	-0,9763	0,2176	6,345	21
S vs. G2/M	1,193	1,719	-0,5265	0,2176	3,422	21
**PRGO powder**						
G0/G1 vs. S	1,847	1,018	0,8291	0,2176	5,388	21
G0/G1 vs. G2/M	1,847	0,5286	1,318	0,2176	8,569	21
S vs. G2/M	1,018	0,5286	0,4894	0,2176	3,181	21
**FRGO powder**						
G0/G1 vs. S	2,08	0,6253	1,455	0,2176	9,454	21
G0/G1 vs. G2/M	2,08	0,4487	1,631	0,2176	10,6	21
S vs. G2/M	0,6253	0,4487	0,1766	0,2176	1,147	21
**GO film**						
G0/G1 vs. S	2,218	0,3042	1,914	0,2176	12,44	21
G0/G1 vs. G2/M	2,218	0,3056	1,912	0,2176	12,43	21
S vs. G2/M	0,3042	0,3056	-0,001374	0,2176	0,008927	21
**PRGO film**						
G0/G1 vs. S	2,151	0,3583	1,792	0,2176	11,65	21
G0/G1 vs. G2/M	2,151	0,443	1,708	0,2176	11,1	21
S vs. G2/M	0,3583	0,443	-0,08474	0,2176	0,5507	21
**FRGO film**						
G0/G1 vs. S	2,285	1,048	1,238	0,2176	8,043	21
G0/G1 vs. G2/M	2,285	0,1431	2,142	0,2176	13,92	21
S vs. G2/M	1,048	0,1431	0,9046	0,2176	5,879	21

Firuge 3 B1

Table Analyzed	Data 1
ANOVA summary	
F	6,238
P value	< 0,0001
P value summary	****
Are differences among means statistically significant? (P < 0.05)	Yes
R square	0,3271
Brown-Forsythe test	
F (DFn, DFd)	2,470 (6, 77)
P value	0,0308
P value summary	*
Significantly different standard deviations? (P < 0.05)	Yes
Bartlett's test	
Bartlett's statistic (corrected)	+infinity
P value	< 0,0001
P value summary	****
Significantly different standard deviations? (P < 0.05)	Yes

ANOVA table	SS	DF	MS	F (DFn, DFd)	P value
Treatment (between columns)	75143	6	12524	F (6, 77) = 6,238	P < 0,0001
Residual (within columns)	154582	77	2008		
Total	229725	83			

Data summary	
Number of treatments (columns)	7
Number of values (total)	84
Number of families	1
Number of comparisons per family	6
Alpha	0,05

Dunnett's multiple comparisons test	Mean Diff,	95% CI of diff,	Significant?	Summary
con**trol** vs. GO **powder**	5,905	-42,11 to 53,92	No	ns
con**trol** vs. PRG**O powder**	66,68	18,66 to 114,7	Yes	*
con**trol** vs. FRG**O powder**	50,42	2,403 to 98,43	Yes	*
con**trol** vs. GO F**ilm**	42,04	-5,977 to 90,05	No	ns
con**trol** vs. PRGGO FILM	84,79	36,77 to 132,8	Yes	****
con**trol** vs. FRGO FI**lm**	69,92	21,90 to 117,9	Yes	**

Test details	Mean 1	Mean 2	Mean Diff,	SE of diff,	n1	n2	q	DF
con**trol** vs. GO **powder**	100	94,09	5,905	18,29	12	12	0,3228	77
con**trol** vs. PRG**O powder**	100	33,32	66,68	18,29	12	12	3,645	77
con**trol** vs. FRG**O powder**	100	49,58	50,42	18,29	12	12	2,756	77
con**trol** vs. GO F**ilm**	100	57,96	42,04	18,29	12	12	2,298	77

con**trol** vs. PRGGO FILM	100	15,21	84,79	18,29	12	12	4,635	77
con**trol** vs. FRGO FI**lm**	100	30,08	69,92	18,29	12	12	3,822	77

Figure 3 B2

Table Analyzed	Data 1				
ANOVA summary					
F	25,78				
P value	< 0,0001				
P value summary	****				
Are differences among means statistically significant? (P < 0.05)	Yes				
R square	0,6676				
Brown-Forsythe test					
F (DFn, DFd)	2,198 (6, 77)				
P value	0,0521				
P value summary	ns				
Significantly different standard deviations? (P < 0.05)	No				
Bartlett's test					
Bartlett's statistic (corrected)	+infinity				
P value	< 0,0001				
P value summary	****				
Significantly different standard deviations? (P < 0.05)	Yes				
ANOVA table	SS	DF	MS	F (DFn, DFd)	P value
Treatment (between columns)	416719	6	69453	F (6, 77) = 25,78	P < 0,0001
Residual (within columns)	207471	77	2694		
Total	624190	83			
Data summary					
Number of treatments (columns)	7				
Number of values (total)	84				

Number of families	1							
Number of comparisons per family	6							
Alpha	0,05							
Dunnett's multiple comparisons test	Mean Diff,	95% CI of diff,	Significant?	Summary				
con**trol** vs. GO **powder**	-41,01	-96,63 to 14,62	No	ns				
con**trol** vs. PRG**O powder**	-154,8	-210,4 to -99,15	Yes	****				
con**trol** vs. FRG**O powder**	-206,7	-262,3 to -151,1	Yes	****				
con**trol** vs. GO Fi**lm**	-120	-175,6 to -64,36	Yes	****				
con**trol** vs. PRGO FILM	-177,7	-233,4 to -122,1	Yes	****				
con**trol** vs. FRGO FI**lm**	-166,1	-221,8 to -110,5	Yes	****				
Test details	Mean 1	Mean 2	Mean Diff,	SE of diff,	n1	n2	q	DF

con**trol** vs. GO **powder**	1	42,01	-41,01	21,19	12	12	1,935	77
con**trol** vs. PRG**O powder**	1	155,8	-154,8	21,19	12	12	7,304	77
con**trol** vs. FRG**O powder**	1	207,7	-206,7	21,19	12	12	9,754	77
con**trol** vs. GO F**ilm**	1	121	-120	21,19	12	12	5,662	77
con**trol** vs. PRGO FILM	1	178,7	-177,7	21,19	12	12	8,387	77
con**trol** vs. FRGO FI**lm**	1	167,1	-166,1	21,19	12	12	7,84	77

Figure 3 B3

Table Analyzed	Data 1				
ANOVA summary					
F	7,048				
P value	< 0,0001				
P value summary	****				
Are differences among means statistically significant? (P < 0.05)	Yes				
R square	0,2871				
Brown-Forsythe test					
F (DFn, DFd)	2,546 (6, 105)				
P value	0,0243				
P value summary	*				
Significantly different standard deviations? (P < 0.05)	Yes				
Bartlett's test					
Bartlett's statistic (corrected)	+infinity				
P value	< 0,0001				
P value summary	****				
Significantly different standard deviations? (P < 0.05)	Yes				
ANOVA table	SS	DF	MS	F (DFn, DFd)	P value
Treatment (between columns)	267047	6	44508	F (6, 105) = 7,048	P < 0,0001
Residual (within columns)	663063	105	6315		
Total	930110	111			

Number of values (total)	112			
Number of comparisons per family	6			
Alpha	0,05			
Dunnett's multiple comparisons test	Mean Diff,	95% CI of diff,	Significant?	Summary
con**trol** vs. GO **powder**	-71,82	-145,1 to 1,489	No	ns
con**trol** vs. PRG**O powder**	28	-45,31 to 101,3	No	ns
con**trol** vs. FRG**O powder**	15,65	-57,66 to 88,96	No	ns
con**trol** vs. GO F**ilm**	43,7	-29,61 to 117,0	No	ns
con**trol** vs. PRGGO FILM	86,82	13,51 to 160,1	Yes	*
con**trol** vs. FRGO FI**lm**	74,23	0,9214 to 147,5	Yes	*

Test details	Mean 1	Mean 2	Mean Diff,	SE of diff,	n1	n2	q	DF
con**trol** vs. GO **powder**	100	171,8	-71,82	28,1	16	16	2,556	105
con**trol** vs. PRG**O powder**	100	72	28	28,1	16	16	0,9967	105
con**trol** vs. FRG**O powder**	100	84,35	15,65	28,1	16	16	0,5571	105
con**trol** vs. GO F**ilm**	100	56,3	43,7	28,1	16	16	1,555	105
con**trol** vs. PRGGO FILM	100	13,18	86,82	28,1	16	16	3,09	105
con**trol** vs. FRGO FI**lm**	100	25,77	74,23	28,1	16	16	2,642	105

Number of comparisons per family	6			
Alpha	0,05			
Dunnett's multiple comparisons test	Mean Diff,	95% CI of diff,	Significant?	Summary
GO **powder** vs. con**trol**	71,82	-1,489 to 145,1	No	ns
GO **powder** vs. PRG**O powder**	99,82	26,51 to 173,1	Yes	**
GO **powder** vs. FRG**O powder**	87,47	14,16 to 160,8	Yes	*
GO **powder** vs. GO F**ilm**	115,5	42,21 to 188,8	Yes	***
GO **powder** vs. PRGGO FILM	158,6	85,33 to 232,0	Yes	****
GO **powder** vs. FRGO FI**lm**	146,1	72,74 to 219,4	Yes	****

Test details	Mean 1	Mean 2	Mean Diff,	SE of diff,	n1	n2	q	DF
GO **powder** vs. con**trol**	171,8	100	71,82	28,1	16	16	2,556	105
GO **powder** vs. PRG**O powder**	171,8	72	99,82	28,1	16	16	3,553	105
GO **powder** vs. FRG**O powder**	171,8	84,35	87,47	28,1	16	16	3,113	105
GO **powder** vs. GO F**ilm**	171,8	56,3	115,5	28,1	16	16	4,112	105
GO **powder** vs. PRGGO FILM	171,8	13,18	158,6	28,1	16	16	5,646	105
GO **powder** vs. FRGO FI**lm**	171,8	25,77	146,1	28,1	16	16	5,198	105

Figure 3 D

Ki67

P value summary- t-test Student	
Significantly different? (P < 0.05)	
GO film**-7 day**<br>vs,<br>Con**trol**	
Unpaired t test	
P value	0,0309
P value summary	*
Significantly different? (P < 0.05)	Yes
One- or two-tailed P value?	Two-tailed
t, df	t=3,266 df=4
How big is the difference?	
Mean ± SEM of column H	1,000 ± 0,0 N=3
Mean ± SEM of column L	0,4975 ± 0,1539 N=3
Difference between means	-0,5025 ± 0,1539
95% confidence interval	-0,9298 to -0,07528
R square	0,7272
Significantly different? (P < 0.05)	
PRGO film**-7 day**<br>vs,<br>Con**trol**	
Unpaired t test	
P value	0,0068
P value summary	**
Significantly different? (P < 0.05)	Yes
One- or two-tailed P value?	Two-tailed
t, df	t=5,136 df=4
How big is the difference?	
Mean ± SEM of column H	1,000 ± 0,0 N=3
Mean ± SEM of column M	0,3148 ± 0,1334 N=3
Difference between means	-0,6852 ± 0,1334
95% confidence interval	-1,056 to -0,3148
R square	0,8683

Significantly different? (P < 0.05)	
FRGO film**-7 day**<br>vs,<br>Con**trol**	
P value	0,0078
P value summary	**
Significantly different? (P < 0.05)	Yes
One- or two-tailed P value?	Two-tailed
t, df	t=4,947 df=4

How big is the difference?	
Mean ± SEM of column H	1,000 ± 0,0 N=3
Mean ± SEM of column N	0,2907 ± 0,1434 N=3
Difference between means	-0,7093 ± 0,1434
95% confidence interval	-1,107 to -0,3112
R square	0,8595
95% confidence interval	-0,03287 to 0,2079
R square	0,5046

Cycline

Anova test

Alpha	0,05			
Dunnett's multiple comparisons test	Mean Diff,	95% CI of diff,	Significant?	Summary
Con**trol** vs. GO p**owder-3 day**	0,2149	-1,515 to 1,945	No	ns
Con**trol** vs. PRGO p**owder-3 day**	-0,1067	-1,836 to 1,623	No	ns
Con**trol** vs. FRGO p**owder-3 day**	-0,241	-1,971 to 1,489	No	ns
Con**trol** vs. GO film**-3 day**	-0,3532	-2,083 to 1,376	No	ns
Con**trol** vs. PRGO film**-3 day**	-0,7399	-2,470 to 0,9897	No	ns
Con**trol** vs. FRGO film**-3 day**	-0,09122	-1,821 to 1,638	No	ns

PH3

ANOVA summary					
F	10,4				
P value	< 0,0001				
P value summary	****				
Are differences among means statistically significant? (P < 0.05)	Yes				
R square	0,8285				
Brown-Forsythe test					
F (DFn, DFd)	0,7368 (13, 28)				
P value	0,7137				
P value summary	ns				
Significantly different standard deviations? (P < 0.05)	No				
Bartlett's test					
Bartlett's statistic (corrected)					
P value					
P value summary					
Significantly different standard deviations? (P < 0.05)					

ANOVA table	SS	DF	MS	F (DFn, DFd)	P value
Treatment (between columns)	75,51	13	5,808	F (13, 28) = 10,40	P < 0,0001
Residual (within columns)	15,63	28	0,5582		
Total	91,14	41			
Data summary					
Number of treatments (columns)	14				
Number of values (total)	42				
Number of families	1				
Number of comparisons per family	13				
Alpha	0,05				
Sidak's multiple comparisons test	Mean Diff,	95% CI of diff,	Significant?	Summary	
Con**trol** vs. GO p**owder-3 day**	-12,53	-23,32 to -1,735	Yes	*	
Con**trol** vs. PRGO p**owder-3 day**	-17,3	-28,10 to -6,513	Yes	***	
Con**trol** vs. FRGO p**owder-3 day**	-18	-28,79 to -7,209	Yes	***	
Con**trol** vs. GO film**-3 day**	-16,74	-27,53 to -5,947	Yes	***	
Con**trol** vs. PRGO film**-3 day**	-15,46	-26,25 to -4,665	Yes	**	
Con**trol** vs. FRGO film**-3 day**	-15,19	-25,98 to -4,397	Yes	**	
Con**trol** vs. Con**trol**	0	-10,79 to 10,79	No	ns	
Con**trol** vs. GO p**owder-7 day**	-0,4308	-11,22 to 10,36	No	ns	
Con**trol** vs. PRGO p**owder-7 day**	-0,4987	-11,29 to 10,29	No	ns	
Con**trol** vs. FRGO p**owder--7 day**	-1,141	-11,93 to 9,650	No	ns	
Con**trol** vs. GO film**-7 day**	-0,4259	-11,22 to 10,37	No	ns	
Con**trol** vs. PRGO film**-7 day**	-0,6065	-11,40 to 10,18	No	ns	
Con**trol** vs. FRGO film**-7 day**	-0,2298	-11,02 to 10,56	No	ns	

Test details	Mean 1	Mean 2	Mean Diff,	SE of diff,	n1	n2	t	DF
Con**trol** vs. GO p**owder-3 day**	1	13,53	-12,53	3,434	3	3	3,648	28
Con**trol** vs. PRGO p**owder-3 day**	1	18,3	-17,3	3,434	3	3	5,039	28
Con**trol** vs. FRGO p**owder-3 day**	1	19	-18	3,434	3	3	5,242	28
Con**trol** vs. GO film**-3 day**	1	17,74	-16,74	3,434	3	3	4,874	28
Con**trol** vs. PRGO film**-3 day**	1	16,46	-15,46	3,434	3	3	4,501	28
Con**trol** vs. FRGO film**-3 day**	1	16,19	-15,19	3,434	3	3	4,423	28
Con**trol** vs. Con**trol**	1	1	0	3,434	3	3	0	28
Con**trol** vs. GO p**owder-7 day**	1	1,431	-0,4308	3,434	3	3	0,1255	28
Con**trol** vs. PRGO p**owder-7 day**	1	1,499	-0,4987	3,434	3	3	0,1452	28
Con**trol** vs. FRGO p**owder--7 day**	1	2,141	-1,141	3,434	3	3	0,3323	28
Con**trol** vs. GO film**-7 day**	1	1,426	-0,4259	3,434	3	3	0,124	28

Control vs. PRGO film-7 day	1	1,607	-0,6065	3,434	3	3	0,1766	28
Control vs. FRGO film-7 day	1	1,23	-0,2298	3,434	3	3	0,06691	28

SP30

ANOVA summary					
F	10,4				
P value	< 0,0001				
P value summary	****				
Are differences among means statistically significant? (P < 0.05)	Yes				
R square	0,8285				
Brown-Forsythe test					
F (DFn, DFd)	0,7368 (13, 28)				
P value	0,7137				
P value summary	ns				
Significantly different standard deviations? (P < 0.05)	No				
Bartlett's test					
Bartlett's statistic (corrected)					
P value					
P value summary					
Significantly different standard deviations? (P < 0.05)					
ANOVA table	SS	DF	MS	F (DFn, DFd)	P value
Treatment (between columns)	75,51	13	5,808	F (13, 28) = 10,40	P < 0,0001
Residual (within columns)	15,63	28	0,5582		
Total	91,14	41			

Alpha	0,05			
Dunnett's multiple comparisons test	Mean Diff,	95% CI of diff,	Significant?	Summary
Control vs. GO powder-3 day	0,06241	-1,760 to 1,885	No	ns
Control vs. PRGO powder-3 day	0,4072	-1,416 to 2,230	No	ns
Control vs. FRGO powder-3 day	0,3215	-1,501 to 2,144	No	ns
Control vs. GO film-3 day	0,4223	-1,400 to 2,245	No	ns
Control vs. PRGO film-3 day	0,05275	-1,770 to 1,875	No	ns
Control vs. FRGO film-3 day	0,2053	-1,617 to 2,028	No	ns
Control vs. Control	0	-1,823 to 1,823	No	ns
Control vs. GO powder-7 day	-0,7898	-2,612 to 1,033	No	ns
Control vs. PRGO powder-7 day	-1,66	-3,483 to 0,1623	No	ns
Control vs. FRGO powder--7 day	-1,558	-3,380 to 0,2651	No	ns
Control vs. GO film-7 day	-2,246	-4,069 to -0,4237	Yes	**
Control vs. PRGO film-7 day	-4,203	-6,025 to -2,380	Yes	****
Control vs. FRGO film-7 day	-2,288	-4,111 to -0,4653	Yes	**

Test details	Mean 1	Mean 2	Mean Diff,	SE of diff,	n1	n2	q	DF
Con**trol** vs. GO p**owder-3 day**	1	0,9376	0,06241	0,6101	3	3	0,1023	28
Con**trol** vs. PRGO p**owder-3 day**	1	0,5928	0,4072	0,6101	3	3	0,6675	28
Con**trol** vs. FRGO p**owder-3 day**	1	0,6785	0,3215	0,6101	3	3	0,527	28
Con**trol** vs. GO film**-3 day**	1	0,5777	0,4223	0,6101	3	3	0,6923	28
Con**trol** vs. PRGO film**-3 day**	1	0,9473	0,05275	0,6101	3	3	0,08646	28
Con**trol** vs. FRGO film**-3 day**	1	0,7947	0,2053	0,6101	3	3	0,3365	28
Con**trol** vs. Con**trol**	1	1	0	0,6101	3	3	0	28
Con**trol** vs. GO p**owder-7 day**	1	1,79	-0,7898	0,6101	3	3	1,295	28
Con**trol** vs. PRGO p**owder-7 day**	1	2,66	-1,66	0,6101	3	3	2,722	28
Con**trol** vs. FRGO p**owder--7 day**	1	2,558	-1,558	0,6101	3	3	2,553	28
Con**trol** vs. GO film**-7 day**	1	3,246	-2,246	0,6101	3	3	3,682	28
Con**trol** vs. PRGO film**-7 day**	1	5,203	-4,203	0,6101	3	3	6,889	28
Con**trol** vs. FRGO film**-7 day**	1	3,288	-2,288	0,6101	3	3	3,751	28

P27

Number of comparisons per family	13			
Alpha	0,05			
Dunnett's multiple comparisons test	Mean Diff,	95% CI of diff,	Significant?	Summary
Con**trol** vs. GO p**owder-3 day**	-0,5082	-2,523 to 1,507	No	ns
Con**trol** vs. PRGO p**owder-3 day**	-0,2959	-2,311 to 1,719	No	ns
Con**trol** vs. FRGO p**owder-3 day**	-0,171	-2,186 to 1,844	No	ns
Con**trol** vs. GO film**-3 day**	-0,3532	-2,368 to 1,662	No	ns
Con**trol** vs. PRGO film**-3 day**	-0,05804	-2,073 to 1,957	No	ns
Con**trol** vs. FRGO film**-3 day**	-0,3765	-2,392 to 1,639	No	ns
Con**trol** vs. Con**trol**	0	-2,015 to 2,015	No	ns
Con**trol** vs. GO p**owder-7 day**	-0,2247	-2,240 to 1,791	No	ns
Con**trol** vs. PRGO p**owder-7 day**	-0,1328	-2,148 to 1,882	No	ns
Con**trol** vs. FRGO p**owder--7 day**	-0,8989	-2,914 to 1,116	No	ns
Con**trol** vs. GO film**-7 day**	-0,5036	-2,519 to 1,512	No	ns
Con**trol** vs. PRGO film**-7 day**	-2,09	-4,105 to -0,07478	Yes	**
Con**trol** vs. FRGO film**-7 day**	-2,543	-4,558 to -0,5278	Yes	**

Test details	Mean 1	Mean 2	Mean Diff,	SE of diff,	n1	n2	q	DF
Con**trol** vs. GO p**owder-3 day**	1	1,508	-0,508	0,6745	3	3	0,7535	28
Con**trol** vs. PRGO p**owder-3 day**	1	1,296	-0,296	0,6745	3	3	0,4387	28
Con**trol** vs. FRGO p**owder-3 day**	1	1,171	-0,171	0,6745	3	3	0,2535	28
Con**trol** vs. GO film**-3 day**	1	1,353	-0,353	0,6745	3	3	0,5236	28
Con**trol** vs. PRGO film**-3 day**	1	1,058	-0,058	0,6745	3	3	0,0861	28
Con**trol** vs. FRGO film**-3 day**	1	1,376	-0,377	0,6745	3	3	0,5582	28
Con**trol** vs. Con**trol**	1	1	0	0,6745	3	3	0	28
Con**trol** vs. GO p**owder-7 day**	1	1,225	-0,225	0,6745	3	3	0,3331	28
Con**trol** vs. PRGO p**owder-7 day**	1	1,133	-0,133	0,6745	3	3	0,197	28
Con**trol** vs. FRGO p**owder--7 day**	1	1,899	-0,899	0,6745	3	3	1,333	28
Con**trol** vs. GO film**-7 day**	1	1,504	-0,504	0,6745	3	3	0,7466	28
Con**trol** vs. PRGO film**-7 day**	1	3,09	-2,09	0,6745	3	3	3,099	28
Con**trol** vs. FRGO film**-7 day**	1	3,543	-2,543	0,6745	3	3	3,77	28

Figure 4.

Control Sample

**time (min)**		**Control**			
**1,39**		28,97	18,79	21,65	20,37
**10,01**		28,52	18,87	21,73	20,13
**18,65**		28,79	19,1	21,7	20,1
**27,29**		28,83	19,29	21,8	20,15
**36,04**	Olig	9,24	7	8,85	7,59
**44,65**		9,29	6,96	8,79	7,88
**53,30**		9,33	6,85	8,66	7,91
**62,04**	FCCP	35,95	27,67	26,84	29,63
**70,69**		34,74	25,76	23,93	25,2
**79,33**		32,96	23,78	22,31	21,52
**88,05**	Ant-Rot	5,76	4,71	6,52	3,53
**96,66**		6,13	5,16	7,02	3,72
**105,29**		5,98	5,02	6,87	3,57
**ndrial oxygen consumption**		5,76	4,71	6,52	3,53
**basal respiration**		23,07	14,58	15,28	16,62
**maximal respiration**		30,19	22,96	20,32	26,1
**proton leak**		3,48	2,14	2,14	4,06
**ATP production**		19,59	12,44	13,14	12,56
**spare respiratory capacity**		7,12	8,38	5,04	9,48
**coupling efficiency**		84,9	85,3	86,0	75,6

Parameter Value	Equation
Non-mitochondrial Oxygen Consumption	Minimum rate measurement after Rotenone/antimycin A injection
Basal Respiration	(Last rate measurement before first injection) – (Non-Mitochondrial Respiration Rate)
Maximal Respiration	(Maximum rate measurement after FCCP injection) – (Non-Mitochondrial Respiration)
H+ (Proton) Leak	(Minimum rate measurement after Oligomycin injection) – (Non-Mitochondrial Respiration
ATP Production	(Last rate measurement before Oligomycin injection) – (Minimum rate measurement after Oligomycin injection)
Spare Respiratory Capacity	(Maximal Respiration) – (Basal Respiration)
Spare Respiratory Capacity as a %	(Maximal Respiration) / (Basal Respiration) × 100
Acute Response	(Last rate measurement before oligomycin Injection) – (Last rate measurement before acute injection)
Coupling Efficiency	ATP Production Rate) / (Basal Respiration Rate) × 100

GO-powder sample

time (min)		GOp						
**1,39**		29,58	22,31	28,18	20,85	21,14	19,55	24,55
**10,01**		28,18	21,45	27,32	21,35	21,79	19,47	24,34
**18,65**		28,07	21,31	27,27	21,38	21,88	19,4	24,34
**27,29**		27,88	21,2	27,02	21,6	22,02	19,46	24,46
**36,04**	Olig	10,71	9,47	11,25	13,42	13,55	12,8	15,15
**44,65**		10,92	9,35	11,74	12,65	13	12,85	15,17
**53,30**		10,86	9,37	12	12,41	12,85	12,59	15
**62,04**	FCCP	28,26	23,76	29,65	20,75	20,58	20,65	30,2
**70,69**		26,95	21,66	28,03	19,79	18,77	18,38	27,99
**79,33**		26,65	21,59	27,82	20,61	18,89	16,95	25,21
**88,05**	Ant-Rot	7,07	6,61	8,15	11,76	11,83	8,32	10,48
**96,66**		7,12	6,64	8,6	11,72	12,1	8,24	10,14
**105,29**		6,85	6,6	8,38	11,68	12,03	7,78	9,96
**nitochondrial oxygen consumption**		6,85	6,6	8,15	11,68	11,83	7,78	9,96
	**basal respiration**	21,03	14,6	18,87	9,92	10,19	11,68	14,5
	**maximal respiration**	21,41	17,16	21,5	9,07	8,75	12,87	20,24
	**proton leak**	3,86	2,75	3,1	0,73	1,02	4,81	5,04
	**ATP production**	17,17	11,85	15,77	12,41	12,85	12,59	15
	**spare respiratory capacity**	0,38	2,56	2,63	-0,85	-1,44	1,19	5,74
	**coupling efficiency**	81,6	81,2	83,6	125,1	126,1	107,8	103,4

Parameter Value	Equation
Non-mitochondrial Oxygen Consumption	Minimum rate measurement after Rotenone/antimycin A injection
Basal Respiration	(Last rate measurement before first injection) – (Non-Mitochondrial Respiration Rate)
Maximal Respiration	(Maximum rate measurement after FCCP injection) – (Non-Mitochondrial Respiration)
H+ (Proton) Leak	(Minimum rate measurement after Oligomycin injection) – (Non-Mitochondrial Respiration
ATP Production	(Last rate measurement before Oligomycin injection) – (Minimum rate measurement after Oligomycin injection)
Spare Respiratory Capacity	(Maximal Respiration) – (Basal Respiration)
Spare Respiratory Capacity as a %	(Maximal Respiration) / (Basal Respiration) × 100
Acute Response	(Last rate measurement before oligomycin Injection) – (Last rate measurement before acute injection)
Coupling Efficiency	ATP Production Rate) / (Basal Respiration Rate) × 100

PRGO -powder sample

time (min)		PRGOp					
**1,39**		25,98	32,6	27,74	28,07	32,29	28,79
**10,01**		25,7	31,66	27,39	27,35	31,57	28,07
**18,65**		25,94	31,6	27,51	27,41	31,47	28,18
**27,29**		26,38	31,8	27,6	27,43	31,57	28,32
**36,04**	Olig	19,83	24,01	21,47	19,75	20,62	18,11
**44,65**		19,78	23,89	21,72	18,6	20,05	17,36
**53,30**		19,41	23,68	21,45	18,35	20,09	17,4
**62,04**	FCCP	33,99	43,3	32,9	39,51	40,54	33,64
**70,69**		35	41,78	33,14	33,62	33,47	28,09
**79,33**		34,61	40,13	35,52	29,39	30,96	26,1
**88,05**	Ant-Rot	17,52	19,84	19,09	12,73	14,75	12,21
**96,66**		17,55	19,95	19,22	13,18	14,79	12,24
**105,29**		16,99	19,69	18,91	13,02	14,75	12,2
**on-mitochondrial oxygen consumption**		16,99	19,69	18,91	12,73	14,75	12,2
	**basal respiration**	9,39	12,11	8,69	14,7	16,82	16,12
	**maximal respiration**	18,01	23,61	16,61	26,78	25,79	21,44
	**proton leak**	2,42	3,99	2,54	5,62	5,3	5,16
	**ATP production**	6,97	8,12	6,15	9,08	11,52	10,96
	**spare respiratory capacity**	8,62	11,5	7,92	12,08	8,97	5,32
	**coupling efficiency**	74,2	67,1	70,8	61,8	68,5	68,0

Parameter Value	Equation
Non-mitochondrial Oxygen Consumption	Minimum rate measurement after Rotenone/antimycin A injection
Basal Respiration	(Last rate measurement before first injection) – (Non-Mitochondrial Respiration Rate)
Maximal Respiration	(Maximum rate measurement after FCCP injection) – (Non-Mitochondrial Respiration)
H+ (Proton) Leak	(Minimum rate measurement after Oligomycin injection) – (Non-Mitochondrial Respiration
ATP Production	(Last rate measurement before Oligomycin injection) – (Minimum rate measurement after Oligomycin injection)
Spare Respiratory Capacity	(Maximal Respiration) – (Basal Respiration)
Spare Respiratory Capacity as a %	(Maximal Respiration) / (Basal Respiration) × 100
Acute Response	(Last rate measurement before oligomycin Injection) – (Last rate measurement before acute injection)
Coupling Efficiency	ATP Production Rate) / (Basal Respiration Rate) × 100

FRGO -powder sample

time (min)		FRGOp			
**1,39**		30	29,9	33,69	25,53
**10,01**		29,39	28,88	32,7	24,96
**18,65**		29,66	28,78	32,8	24,91
**27,29**		29,57	28,92	32,95	24,82
**36,04**	Olig	20,38	19,81	21,99	18,42
**44,65**		20,05	19,57	21,7	17,1
**53,30**		19,41	19,26	21,35	16,78
**62,04**	FCCP	40,75	41,58	42,79	36,31
**70,69**		39,18	41,37	44,51	34,88
**79,33**		37,12	40,72	44,63	32,49
**88,05**	Ant-Rot	18,23	17,26	18,85	12,72
**96,66**		18,24	17,47	19,02	12,75
**105,29**		17,64	17,04	18,79	12,38
**chondrial oxygen consumption**		17,64	17,04	18,79	12,38
**basal respiration**		11,93	11,88	14,16	12,44
**maximal respiration**		23,11	24,54	25,84	23,93
	**proton leak**	1,77	2,22	2,56	4,4
**ATP production**		10,16	9,66	11,6	8,04
**spare respiratory capacity**		11,18	12,66	11,68	11,49
**coupling efficiency**		85,2	81,3	81,9	64,6

Parameter Value	Equation
Non-mitochondrial Oxygen Consumption	Minimum rate measurement after Rotenone/antimycin A injection
Basal Respiration	(Last rate measurement before first injection) – (Non-Mitochondrial Respiration Rate)
Maximal Respiration	(Maximum rate measurement after FCCP injection) – (Non-Mitochondrial Respiration)
H+ (Proton) Leak	(Minimum rate measurement after Oligomycin injection) – (Non-Mitochondrial Respiration
ATP Production	(Last rate measurement before Oligomycin injection) – (Minimum rate measurement after Oligomycin injection)
Spare Respiratory Capacity	(Maximal Respiration) – (Basal Respiration)
Spare Respiratory Capacity as a %	(Maximal Respiration) / (Basal Respiration) × 100
Acute Response	(Last rate measurement before oligomycin Injection) – (Last rate measurement before acute injection)
Coupling Efficiency	ATP Production Rate) / (Basal Respiration Rate) × 100

SAMPLE GO FILM

SAMP

time (min)		GO-film				
**1,39**		29,98	31,06	31,94	30,76	22,4
**10,01**		29,32	30,11	33,13	31,22	22,57
**18,65**		29,42	30,18	33,8	31,28	22,55
**27,29**		29,31	30,21	33,85	31,38	22,69
**36,04**	Olig	19,23	20,41	23,45	20,22	13,66
**44,65**		19,17	20,09	23,54	20,57	13,76
**53,30**		19,02	20,19	23,27	20,57	13,82
**62,04**	FCCP	35,82	35,43	34,98	38,28	30,01
**70,69**		33,92	34,39	34,46	37,17	28,35
**79,33**		33,06	32,12	35,16	37,07	27,59
**88,05**	Ant-Rot	15,4	17,38	14,42	18,43	10,04
**96,66**		16,07	18,08	14,98	19,42	10,3
**105,29**		16	18,22	14,51	19,4	10,29
**n-mitochondrial oxygen consumption**		15,4	17,38	14,42	18,43	10,04
	**basal respiration**	13,91	12,83	19,43	12,95	12,65
	**maximal respiration**	20,42	18,05	20,74	19,85	19,97
	**proton leak**	3,62	2,71	8,85	1,79	3,62
	**ATP production**	10,29	10,12	10,58	11,16	9,03
	**spare respiratory capacity**	6,51	5,22	1,31	6,90	7,32
	**coupling efficiency**	74,0	78,9	54,5	86,2	71,4

Parameter Value	Equation
Non-mitochondrial Oxygen Consumption	Minimum rate measurement after Rotenone/antimycin A injection
Basal Respiration	(Last rate measurement before first injection) – (Non-Mitochondrial Respiration Rate)
Maximal Respiration	(Maximum rate measurement after FCCP injection) – (Non-Mitochondrial Respiration)
H+ (Proton) Leak	(Minimum rate measurement after Oligomycin injection) – (Non-Mitochondrial Respiration
ATP Production	(Last rate measurement before Oligomycin injection) – (Minimum rate measurement after Oligomycin injection)
Spare Respiratory Capacity	(Maximal Respiration) – (Basal Respiration)
Spare Respiratory Capacity as a %	(Maximal Respiration) / (Basal Respiration) × 100
Acute Response	(Last rate measurement before oligomycin Injection) – (Last rate measurement before acute injection)
Coupling Efficiency	ATP Production Rate) / (Basal Respiration Rate) × 100

SAMPLE PRGO FILM

time (min)		PRGO-film		
**1,39**		29,12	25,96	26,54
**10,01**		28,54	26,17	26,6
**18,65**		28,64	26,31	26,74
**27,29**		28,55	26,53	27
**36,04**	Olig	15,2	15,7	18,47
**44,65**		15,41	15,78	17,79
**53,30**		15,44	15,69	17,63
**62,04**	FCCP	33,79	40,32	31,48
**70,69**		29,72	34,35	27,78
**79,33**		29,86	30,45	25,3
**88,05**	Ant-Rot	11,89	10,31	13,28
**96,66**		12,47	10,66	13,29
**105,29**		12,29	10,31	12,99
**-mitochondrial oxygen consumption**		11,89	10,31	12,99
	**basal respiration**	16,66	16,22	14,01
	**maximal respiration**	21,9	30,01	18,49
	**proton leak**	3,31	5,38	4,64
	**ATP production**	13,35	10,84	9,37
	**spare respiratory capacity**	5,24	13,79	4,48
	**coupling efficiency**	80,1	66,8	66,9

Parameter Value	Equation
Non-mitochondrial Oxygen Consumption	Minimum rate measurement after Rotenone/antimycin A injection
Basal Respiration	(Last rate measurement before first injection) – (Non-Mitochondrial Respiration Rate)
Maximal Respiration	(Maximum rate measurement after FCCP injection) – (Non-Mitochondrial Respiration)
H+ (Proton) Leak	(Minimum rate measurement after Oligomycin injection) – (Non-Mitochondrial Respiration
ATP Production	(Last rate measurement before Oligomycin injection) – (Minimum rate measurement after Oligomycin injection)
Spare Respiratory Capacity	(Maximal Respiration) – (Basal Respiration)
Spare Respiratory Capacity as a %	(Maximal Respiration) / (Basal Respiration) × 100
Acute Response	(Last rate measurement before oligomycin Injection) – (Last rate measurement before acute injection)
Coupling Efficiency	ATP Production Rate) / (Basal Respiration Rate) × 100

SAMPLE FRGO -FILM

time (min)		FRGO-film			
**1,39**		22,6	21,88	17,54	17,27
**10,01**		21,97	22,07	17,65	17,15
**18,65**		22,03	21,99	17,72	17,22
**27,29**		22,04	22,13	17,71	17,23
**36,04**	Olig	16,06	16,48	12,79	11,79
**44,65**		16,29	16,47	12,96	11,14
**53,30**		16,18	16,35	12,87	10,85
**62,04**	FCCP	26,54	27,5	21,38	22,49
**70,69**		25,33	27,31	20,84	20,07
**79,33**		24,12	26,53	20,5	18,5
**88,05**	Ant-Rot	13,72	13,9	11,28	7,59
**96,66**		14,06	13,85	11,3	7,59
**105,29**		13,85	13,76	11,2	7,42
**non-mitochondrial oxygen consumption**		13,72	13,76	11,2	7,42
	**basal respiration**	8,32	8,37	6,51	9,81
	**maximal respiration**	12,82	13,74	10,18	15,07
	**proton leak**	2,34	2,59	1,67	3,43
	**ATP production**	5,98	5,78	4,84	6,38
	**spare respiratory capacity**	4,50	5,37	3,67	5,26
	**coupling efficiency**	71,9	69,1	74,3	65,0

Parameter Value	Equation
Non-mitochondrial Oxygen Consumption	Minimum rate measurement after Rotenone/antimycin A injection
Basal Respiration	(Last rate measurement before first injection) – (Non-Mitochondrial Respiration Rate)
Maximal Respiration	(Maximum rate measurement after FCCP injection) – (Non-Mitochondrial Respiration)
H+ (Proton) Leak	(Minimum rate measurement after Oligomycin injection) – (Non-Mitochondrial Respiration
ATP Production	(Last rate measurement before Oligomycin injection) – (Minimum rate measurement after Oligomycin injection)
Spare Respiratory Capacity	(Maximal Respiration) – (Basal Respiration)
Spare Respiratory Capacity as a %	(Maximal Respiration) / (Basal Respiration) × 100
Acute Response	(Last rate measurement before oligomycin Injection) – (Last rate measurement before acute injection)
Coupling Efficiency	ATP Production Rate) / (Basal Respiration Rate) × 100

Figure 6B

Test T-Student

Control VS GO powderr	no significant	P value	Mean1	Mean2	Difference	SE of difference	t ratio	df
		0,425158	1	1,39427	-0,39427	0,460907	0,855413	6
Control vs PRGOpowder	Significcant p<0,05	P value	Mean1	Mean2	Difference	SE of difference	t ratio	df
	**	0,000463	1	3,05156	-2,05156	0,297917	6,88633	6
Control vs FRGOpowder	Significcant p<0,05	P value	Mean1	Mean2	Difference	SE of difference	t ratio	df
	*	0,039965	1	2,80309	-1,80309	0,690077	2,61289	6
Control vs GO film	Significcant p<0,05	P value	Mean1	Mean2	Difference	SE of difference	t ratio	df
	*	0,016662	1	2,15051	-1,15051	0,278761	4,12722	6
Control vs PRGO film	Significcant p<0,05	P value	Mean1	Mean2	Difference	SE of difference	t ratio	df
	***	< 0,0001	1	3,12916	-2,12916	0,167179	12,7358	6
Control vs FRGO-F	Significcant p<0,05	P value	Mean1	Mean2	Difference	SE of difference	t ratio	df
	***	< 0,0001	1	2,64427	-1,64427	0,177546	9,26105	6

Figure 7A

Two-way ANOVA	Ordinary				
Alpha	0,05				
Source of Variation	% of total variation	P value	P value summary	Significant?	
Interaction	42,58	< 0,0001	****	Yes	
Row Factor	9,214	< 0,0001	****	Yes	
Column Factor	47,88	< 0,0001	****	Yes	
ANOVA table	SS	DF	MS	F (DFn, DFd)	P value
Interaction	4818	12	401,5	F (12, 42) = 464,1	P < 0,0001
Row Factor	1043	2	521,3	F (2, 42) = 602,6	P < 0,0001
Column Factor	5419	6	903,1	F (6, 42) = 1044	P < 0,0001
Residual	36,34	42	0,8652		
Number of missing values	0				
Number of comparisons per family	21				
Alpha	0,05				
Tukey's multiple comparisons test					
**Th**	Mean Diff,	95% CI of diff,	Significant?	Summary	
Control vs. GO powder	-1,12	-3,471 to 1,231	No	ns	
Control vs. PRGO powder	-1,534	-3,885 to 0,8170	No	ns	
Control vs. FRGO powder	-6,867	-9,218 to -4,516	Yes	****	
Control vs. GO fIlm	-1,606	-3,957 to 0,7450	No	ns	
Control vs. PRGO film	-18,93	-21,28 to -16,58	Yes	****	
Control vs. FRGOfilm	-1,682	-4,033 to 0,6693	No	ns	
GO powder vs. PRGO powder	-0,4141	-2,765 to 1,937	No	ns	
GO powder vs. FRGO powder	-5,747	-8,098 to -3,396	Yes	****	
GO powder vs. GO fIlm	-0,4861	-2,837 to 1,865	No	ns	
GO powder vs. PRGO film	-17,81	-20,16 to -15,46	Yes	****	
GO powder vs. FRGOfilm	-0,5619	-2,913 to 1,789	No	ns	
PRGO powder vs. FRGO powder	-5,333	-7,684 to -2,982	Yes	****	
PRGO powder vs. GO fIlm	-0,07198	-2,423 to 2,279	No	ns	

PRGO powder vs. PRGO film	-17,4	-19,75 to -15,05	Yes	****
PRGO powder vs. FRGOfilm	-0,1477	-2,499 to 2,203	No	ns
FRGO powder vs. GO fIlm	5,261	2,910 to 7,612	Yes	****
FRGO powder vs. PRGO film	-12,07	-14,42 to -9,715	Yes	****
FRGO powder vs. FRGOfilm	5,185	2,834 to 7,536	Yes	****
GO fIlm vs. PRGO film	-17,33	-19,68 to -14,98	Yes	****
GO fIlm vs. FRGOfilm	-0,07574	-2,427 to 2,275	No	ns
PRGO film vs. FRGOfilm	17,25	14,90 to 19,60	Yes	****
**Nr4a2/Nurr1**	Mean Diff,	95% CI of diff,	Significant?	Summary
Control vs. GO powder	-1,355	-3,706 to 0,9959	No	ns
Control vs. PRGO powder	-4,5	-6,851 to -2,149	Yes	****
Control vs. FRGO powder	-1,456	-3,807 to 0,8954	No	ns
Control vs. GO fIlm	-2,228	-4,579 to 0,1228	No	ns
Control vs. PRGO film	-3,69	-6,041 to -1,339	Yes	***
Control vs. FRGOfilm	-3,649	-6,000 to -1,298	Yes	***
GO powder vs. PRGO powder	-3,145	-5,496 to -0,7941	Yes	**
GO powder vs. FRGO powder	-0,1006	-2,452 to 2,250	No	ns
GO powder vs. GO fIlm	-0,8731	-3,224 to 1,478	No	ns
GO powder vs. PRGO film	-2,335	-4,686 to 0,01618	No	ns
GO powder vs. FRGOfilm	-2,294	-4,645 to 0,05682	No	ns
PRGO powder vs. FRGO powder	3,045	0,6936 to 5,396	Yes	**
PRGO powder vs. GO fIlm	2,272	-0,07897 to 4,623	No	ns
PRGO powder vs. PRGO film	0,8103	-1,541 to 3,161	No	ns
PRGO powder vs. FRGOfilm	0,8509	-1,500 to 3,202	No	ns
FRGO powder vs. GO fIlm	-0,7725	-3,124 to 1,578	No	ns
FRGO powder vs. PRGO film	-2,234	-4,585 to 0,1167	No	ns
FRGO powder vs. FRGOfilm	-2,194	-4,545 to 0,1574	No	ns
GO fIlm vs. PRGO film	-1,462	-3,813 to 0,8893	No	ns
GO fIlm vs. FRGOfilm	-1,421	-3,772 to 0,9299	No	ns

PRGO film vs. FRGOfilm	0,04064	-2,310 to 2,392	No	ns
**Dat**	Mean Diff,	95% CI of diff,	Significant?	Summary
Control vs. GO powder	-1,048	-3,399 to 1,303	No	ns
Control vs. PRGO powder	-1,167	-3,518 to 1,184	No	ns
Control vs. FRGO powder	-16,53	-18,88 to -14,17	Yes	****
Control vs. GO fIlm	-1,615	-3,966 to 0,7361	No	ns
Control vs. PRGO film	-61,96	-64,31 to -59,61	Yes	****
Control vs. FRGOfilm	-1,018	-3,369 to 1,333	No	ns
GO powder vs. PRGO powder	-0,1196	-2,471 to 2,231	No	ns
GO powder vs. FRGO powder	-15,48	-17,83 to -13,13	Yes	****
GO powder vs. GO fIlm	-0,5674	-2,918 to 1,784	No	ns
GO powder vs. PRGO film	-60,91	-63,26 to -58,56	Yes	****
GO powder vs. FRGOfilm	0,02989	-2,321 to 2,381	No	ns
PRGO powder vs. FRGO powder	-15,36	-17,71 to -13,01	Yes	****
PRGO powder vs. GO fIlm	-0,4477	-2,799 to 1,903	No	ns
PRGO powder vs. PRGO film	-60,79	-63,14 to -58,44	Yes	****
PRGO powder vs. FRGOfilm	0,1495	-2,201 to 2,501	No	ns
FRGO powder vs. GO fIlm	14,91	12,56 to 17,26	Yes	****
FRGO powder vs. PRGO film	-45,43	-47,78 to -43,08	Yes	****
FRGO powder vs. FRGOfilm	15,51	13,16 to 17,86	Yes	****
GO fIlm vs. PRGO film	-60,34	-62,70 to -57,99	Yes	****
GO fIlm vs. FRGOfilm	0,5973	-1,754 to 2,948	No	ns
PRGO film vs. FRGOfilm	60,94	58,59 to 63,29	Yes	****

Test details	Mean 1	Mean 2	Mean Diff,	SE of diff,	N1	N2	q	DF
**Th**								
Control vs. GO powder	1	2,1	-1	0,8	3	3	2,1	42
Control vs. PRGO powder	1	2,5	-2	0,8	3	3	2,9	42
Control vs. FRGO powder	1	7,9	-7	0,8	3	3	13	42
Control vs. GO fIlm	1	2,6	-2	0,8	3	3	3	42
Control vs. PRGO film	1	20	-19	0,8	3	3	35	42
Control vs. FRGOfilm	1	2,7	-2	0,8	3	3	3,1	42
GO powder vs. PRGO powder	2,1	2,5	-0	0,8	3	3	0,8	42
GO powder vs. FRGO powder	2,1	7,9	-6	0,8	3	3	11	42
GO powder vs. GO fIlm	2,1	2,6	-0	0,8	3	3	0,9	42
GO powder vs. PRGO film	2,1	20	-18	0,8	3	3	33	42
GO powder vs. FRGOfilm	2,1	2,7	-1	0,8	3	3	1	42
PRGO powder vs. FRGO powder	2,5	7,9	-5	0,8	3	3	9,9	42
PRGO powder vs. GO fIlm	2,5	2,6	-0	0,8	3	3	0,1	42
PRGO powder vs. PRGO film	2,5	20	-17	0,8	3	3	32	42
PRGO powder vs. FRGOfilm	2,5	2,7	-0	0,8	3	3	0,3	42
FRGO powder vs. GO fIlm	7,9	2,6	5,3	0,8	3	3	9,8	42
FRGO powder vs. PRGO film	7,9	20	-12	0,8	3	3	22	42
FRGO powder vs. FRGOfilm	7,9	2,7	5,2	0,8	3	3	9,7	42
GO fIlm vs. PRGO film	2,6	20	-17	0,8	3	3	32	42
GO fIlm vs. FRGOfilm	2,6	2,7	-0	0,8	3	3	0,1	42
PRGO film vs. FRGOfilm	20	2,7	17	0,8	3	3	32	42
**Nr4a2/Nurr**								
Control vs. GO powder	1	2,4	-1	0,8	3	3	2,5	42
Control vs. PRGO powder	1	5,5	-5	0,8	3	3	8,4	42
Control vs. FRGO powder	1	2,5	-1	0,8	3	3	2,7	42
Control vs. GO fIlm	1	3,2	-2	0,8	3	3	4,1	42
Control vs. PRGO film	1	4,7	-4	0,8	3	3	6,9	42
Control vs. FRGOfilm	1	4,6	-4	0,8	3	3	6,8	42
GO powder vs. PRGO powder	2,4	5,5	-3	0,8	3	3	5,9	42
GO powder vs. FRGO powder	2,4	2,5	-0	0,8	3	3	0,2	42
GO powder vs. GO fIlm	2,4	3,2	-1	0,8	3	3	1,6	42
GO powder vs. PRGO film	2,4	4,7	-2	0,8	3	3	4,3	42
GO powder vs. FRGOfilm	2,4	4,6	-2	0,8	3	3	4,3	42
PRGO powder vs. FRGO powder	5,5	2,5	3	0,8	3	3	5,7	42
PRGO powder vs. GO fIlm	5,5	3,2	2,3	0,8	3	3	4,2	42
PRGO powder vs. PRGO film	5,5	4,7	0,8	0,8	3	3	1,5	42
PRGO powder vs. FRGOfilm	5,5	4,6	0,9	0,8	3	3	1,6	42
FRGO powder vs. GO fIlm	2,5	3,2	-1	0,8	3	3	1,4	42
FRGO powder vs. PRGO film	2,5	4,7	-2	0,8	3	3	4,2	42

FRGO powder vs. FRGOfilm	2,5	4,6	-2	0,8	3	3	4,1	42
GO fIlm vs. PRGO film	3,2	4,7	-1	0,8	3	3	2,7	42
GO fIlm vs. FRGOfilm	3,2	4,6	-1	0,8	3	3	2,6	42
PRGO film vs. FRGOfilm	4,7	4,6	0	0,8	3	3	0,1	42
**Dat**								
Control vs. GO powder	1	2	-1	0,8	3	3	2	42
Control vs. PRGO powder	1	2,2	-1	0,8	3	3	2,2	42
Control vs. FRGO powder	1	18	-17	0,8	3	3	31	42
Control vs. GO fIlm	1	2,6	-2	0,8	3	3	3	42
Control vs. PRGO film	1	63	-62	0,8	3	3	115	42
Control vs. FRGOfilm	1	2	-1	0,8	3	3	1,9	42
GO powder vs. PRGO powder	2	2,2	-0	0,8	3	3	0,2	42
GO powder vs. FRGO powder	2	18	-15	0,8	3	3	29	42
GO powder vs. GO fIlm	2	2,6	-1	0,8	3	3	1,1	42
GO powder vs. PRGO film	2	63	-61	0,8	3	3	113	42
GO powder vs. FRGOfilm	2	2	0	0,8	3	3	0,1	42
PRGO powder vs. FRGO powder	2,2	18	-15	0,8	3	3	29	42
PRGO powder vs. GO fIlm	2,2	2,6	-0	0,8	3	3	0,8	42
PRGO powder vs. PRGO film	2,2	63	-61	0,8	3	3	113	42
PRGO powder vs. FRGOfilm	2,2	2	0,1	0,8	3	3	0,3	42
FRGO powder vs. GO fIlm	18	2,6	15	0,8	3	3	28	42
FRGO powder vs. PRGO film	18	63	-45	0,8	3	3	85	42
FRGO powder vs. FRGOfilm	18	2	16	0,8	3	3	29	42
GO fIlm vs. PRGO film	2,6	63	-60	0,8	3	3	112	42
GO fIlm vs. FRGOfilm	2,6	2	0,6	0,8	3	3	1,1	42
PRGO film vs. FRGOfilm	63	2	61	0,8	3	3	114	42

Figure 7B

Two-way RM ANOVA	Matching: Both factors				
Alpha	0,05				
Source of Variation	% of total variation	P value	P value summary	Significant?	
Row Factor	86,25	< 0,0001	****	Yes	
Column Factor	3,145	< 0,0001	****	Yes	
Interaction: Row Factor x Column Factor	7,892	< 0,0001	****	Yes	
Interaction: Row Factor x Subjects	0,4942				
Interaction: Column Factor x Subjects	0,116				
Subjects	0,1366				
ANOVA table	SS	DF	MS	F (DFn, DFd)	P value
Row Factor	33,51	6	5,584	F (6, 12) = 349,0	P < 0,0001
Column Factor	1,222	3	0,4072	F (3, 6) = 54,20	P < 0,0001
Interaction: Row Factor x Column Factor	3,066	18	0,1703	F (18, 36) = 8,023	P < 0,0001
Interaction: Row Factor x Subjects	0,192	12	0,016		
Interaction: Column Factor x Subjects	0,04508	6	0,00751		
Subjects	0,05308	2	0,02654		
Residual	0,7643	36	0,02123		

Tukey's multiple comparisons test				
Tubb3	Mean Diff,	95% CI of diff,	Significant?	Summary
Control vs. GO powder	-1,657	-2,028 to -1,285	Yes	****
Control vs. PRGO powder	-1,833	-2,205 to -1,462	Yes	****
Control vs. FRGO powder	-2,013	-2,385 to -1,642	Yes	****
Control vs. GO film	-2,104	-2,475 to -1,733	Yes	****
Control vs. PRGO film	-2,183	-2,554 to -1,811	Yes	****
Control vs. FRGO film	-1,869	-2,240 to -1,498	Yes	****
Pitx3	Mean Diff,	95% CI of diff,	Significant?	Summary
Control vs. GO powder	-1,921	-2,292 to -1,549	Yes	****
Control vs. PRGO powder	-1,642	-2,013 to -1,270	Yes	****
Control vs. FRGO powder	-2,06	-2,431 to -1,689	Yes	****
Control vs. GO film	-1,367	-1,738 to -0,9956	Yes	****
Control vs. PRGO film	-1,441	-1,813 to -1,070	Yes	****
Control vs. FRGO film	-1,269	-1,640 to -0,8973	Yes	****
Limx1a	Mean Diff,	95% CI of diff,	Significant?	Summary
Control vs. GO powder	-2,048	-2,419 to -1,676	Yes	****
Control vs. PRGO powder	-1,522	-1,893 to -1,151	Yes	****
Control vs. FRGO powder	-2,077	-2,448 to -1,705	Yes	****
Control vs. GO film	-1,263	-1,634 to -0,8915	Yes	****
Control vs. PRGO film	-1,364	-1,735 to -0,9924	Yes	****
Control vs. FRGO film	-1,249	-1,621 to -0,8781	Yes	****
Limx1b	Mean Diff,	95% CI of diff,	Significant?	Summary
Control vs. GO powder	-2,127	-2,498 to -1,756	Yes	****
Control vs. PRGO powder	-1,905	-2,276 to -1,534	Yes	****
Control vs. FRGO powder	-2,027	-2,398 to -1,655	Yes	****
Control vs. GO film	-1,329	-1,700 to -0,9572	Yes	****
Control vs. PRGO film	-1,363	-1,734 to -0,9913	Yes	****
Control vs. FRGO film	-1,321	-1,692 to -0,9498	Yes	****

Figure 7C

ANOVA ONE WAY	
Table Analyzed	Data 1
Repeated measures ANOVA summary	
Assume sphericity?	No
F	6,296
P value	0,0311
P value summary	*
Statistically significant (P < 0.05)?	Yes
Geisser-Greenhouse's epsilon	0,3475
R square	0,6773

		SIGNIFCANT?	P value	Mean 1	Mean 2	Difference	SE of difference	t ratio	df
GOP	TH		0,1705	1	9,2133	-8,21327	5,27569	1,55682	6
PRGOP	TH	**	0,0001	1	21,436	-20,4364	2,40112	8,5112	6
FRGOP	TH	*	0,0012	1	16,802	-15,802	2,74755	5,75128	6
GOF	TH	*	0,0508	1	11,515	-10,5152	4,31797	2,43522	6
PRGO-F	TH	**	0,0007	1	35,625	-34,6245	5,37637	6,44012	6
FROG-F	TH		0,0598	1	12,768	-11,7675	5,08189	2,31559	6

Figure 7D

ANOVA summary<br><br>syantobrevin								
prgo-fiilm	P value	Mean1	Mean2	Difference	SE of difference	t ratio	df	
**SINAPTOBREVIN-**2	0,00017	0,18423	1026,78	-1026,6	76,0941	13,4911	4	
Number of families	1							
Number of comparisons per family	6							
Alpha	0,05							
Dunnett's multiple comparisons test	Mean Diff,	95% CI of diff,	Significant?	Summary				
CONTROL vs. GO powder	-73,51	-300,5 to 153,5	No	ns				
CONTROL vs. PRGO powder	-186,6	-651,9 to 278,8	No	ns				
CONTROL vs. FRGO powder	-408,4	-2293 to 1477	No	ns				
CONTROL vs. GO film	-226,7	-972,7 to 519,4	No	ns				
CONTROL vs. PRGO film	-1027	-1570 to -483,2	Yes	**				
CONTROL vs. FRGO film	-139,6	-575,1 to 295,9	No	ns				
Test details	Mean 1	Mean 2	Mean Diff,	SE of diff,	n1	n2	q	DF
CONTROL vs. GO powder	0,1842	73,69	-73,51	31,86	3	3	2,307	2
CONTROL vs. PRGO powder	0,1842	186,8	-186,6	65,31	3	3	2,857	2
CONTROL vs. FRGO powder	0,1842	408,6	-408,4	264,6	3	3	1,544	2
CONTROL vs. GO film	0,1842	226,9	-226,7	104,7	3	3	2,165	2
CONTROL vs. PRGO film	0,1842	1027	-1027	76,26	3	3	13,46	2
CONTROL vs. FRGO film	0,1842	139,8	-139,6	61,12	3	3	2,284	2
DAT								
Number of families	1							
Number of comparisons per family	6							
Alpha	0,05							
Dunnett's multiple comparisons test	Mean Diff,	95% CI of diff,	Significant?	Summary				
Con**trol** vs. GO p**owder**	-1,382	-15,41 to 12,65	No	ns				
Con**trol** vs. PRGO p**owder**	-5,533	-19,56 to 8,496	No	ns				
Con**trol** vs. FRGO p**owder**	-0,1535	-14,18 to 13,88	No	ns				
Con**trol** vs. GO film	-6,521	-20,55 to 7,508	No	ns				
Con**trol** vs. PRGO film	-19,28	-33,30 to -5,247	Yes	**				
Con**trol** vs. FRGO film	0	-14,03 to 14,03	No	ns				
Test details	Mean 1	Mean 2	Mean Diff,	SE of diff,	n1	n2	q	DF
Con**trol** vs. GO p**owder**	1E-08	1,382	-1,382	4,817	3	3	0,2869	14
Con**trol** vs. PRGO p**owder**	1E-08	5,533	-5,533	4,817	3	3	1,149	14
Con**trol** vs. FRGO p**owder**	1E-08	0,1535	-0,1535	4,817	3	3	0,03186	14
Con**trol** vs. GO film	1E-08	6,521	-6,521	4,817	3	3	1,354	14
Con**trol** vs. PRGO film	1E-08	19,28	-19,28	4,817	3	3	4,002	14

Control vs. FRGO film	1E-08	1E-08	0	4,817	3	3	0	14
Table Analyzed	Data 1							
Column F	PRGO film							
vs.	vs,							
Column A	Control							
Unpaired t test								
P value	0,0469							
P value summary	*							
Significantly different? (P < 0.05)	Yes							
One- or two-tailed P value?	Two-tailed							
t, df	t=2,839 df=4							
How big is the difference?								
Mean ± SEM of column A	1,000e-008 ± 0,0 N=3							
Mean ± SEM of column F	19,28 ± 6,790 N=3							
Difference between means	19,28 ± 6,790							
95% confidence interval	0,4234 to 38,13							
R square	0,6683							
F test to compare variances								
F,DFn, Dfd								
P value								
P value summary								
Significantly different? (P < 0.05)								
SYNAPTOFISIN								
C-GOP	Significant?	P value	Mean1	Mean2	Difference	SE of difference	t ratio	df
SINAPTOPHYSIN		0,22743	0,11667	0,51161	-0,3949	0,27726	1,42444	4
C-PRGOP	Significant?	P value	Mean1	Mean2	Difference	SE of difference	t ratio	df
SINAPTOPHYSIN	*	0,04565	0,11667	5,74001	-5,6233	1,96202	2,86609	4
C-FRGOP	Significant?	P value	Mean1	Mean2	Difference	SE of difference	t ratio	df
SINAPTOPHYSIN		0,76745	0,11667	0,15894	-0,0423	0,13355	0,31651	4
C-GOF	Significant?	P value	Mean1	Mean2	Difference	SE of difference	t ratio	df
SINAPTOPHYSIN	*	0,04973	0,11667	2,99129	-2,8746	1,03336	2,78184	4
PRGO-FILM	Significant?	P value	Mean1	Mean2	Difference	SE of difference	t ratio	df
SINAPTOPHYSIN	*	0,04565	0,11667	5,74001	-5,6233	1,96202	2,86609	4
FRGO--FILM	Significant?	P value	Mean1	Mean2	Difference	SE of difference	t ratio	df
SINAPTOPHYSIN		0,30115	0,11667	0,6505	-0,5338	0,45	1,18631	4

Table Analyzed 1

ANOVA table	SS	DF	MS	F (DFn, DFd)	P value

Treatment (between columns)	3851	6	641,9	F (1,008, 2,017) = 7,214	P = 0,1143
Individual (between rows)	585,2	2	292,6	F (2, 12) = 3,288	P = 0,0727
Residual (random)	1068	12	88,98		
Total	5504	20			

Alpha	0,05							
GIRK2	GIRK3	GIRK4	GIRK5	GIRK6				
Dunnett's multiple comparisons test	Mean Diff,	95% CI of diff,	Significant?	Summary				
Con**trol** vs. GO p**owder**	-0,08088	-0,6928 to 0,5310	No	ns				
Con**trol** vs. PRGO p**owder**	-21,04	-88,13 to 46,05	No	ns				
Con**trol** vs. FRGO p**owder**	-0,02006	-0,1986 to 0,1585	No	ns				
Con**trol** vs. GO film	-17,96	-111,7 to 75,81	No	ns				
Con**trol** vs. PRGO film	-36,55	-62,84 to -10,26	Yes	*				
Con**trol** vs. FRGO film	0			ns				
Test details	Mean 1	Mean 2	Mean Diff,	SE of diff,	n1	n2	q	DF
Con**trol** vs. GO p**owder**	0,005	0,08588	-0,08088	0,08588	3	3	0,9418	2
Con**trol** vs. PRGO p**owder**	0,005	21,05	-21,04	9,416	3	3	2,235	2
Con**trol** vs. FRGO p**owder**	0,005	0,02506	-0,02006	0,02506	3	3	0,8005	2
Con**trol** vs. GO film	0,005	17,97	-17,96	13,16	3	3	1,365	2
Con**trol** vs. PRGO film	0,005	36,55	-36,55	3,69	3	3	9,904	2
Con**trol** vs. FRGO film	0	0	0	0	3	3		

Fi

Figure 8

Figure 8 A

α -Synuclein

ANOVA summary

F	28,62
P value	< 0,0001
P value summary	****
Are differences among means statistically significant? (P < 0.05)	Yes
R square	0,8388
Brown-Forsythe test	
F (DFn, DFd)	1,853 (6, 33)
P value	0,119
P value summary	ns
Significantly different standard deviations? (P < 0.05)	No
Bartlett's test	
Bartlett's statistic (corrected)	+infinity
P value	< 0,0001
P value summary	****
Significantly different standard deviations? (P < 0.05)	Yes

**ANOVA table**	SS	DF	MS	F (DFn, DFd)	P value
Treatment (between columns)	2,877	6	0,4795	F (6, 33) = 28,62	P < 0,0001
Residual (within columns)	0,5529	33	0,01675		
Total	3,43	39			

Sidak's multiple comparisons test	Mean Diff,	95% CI of diff,	Significant?	Summary
control vs. GO powder	0,2515	0,04241 to 0,4607	Yes	*
control vs. PRGO powder	0,2971	0,08795 to 0,5062	Yes	*
control vs. FRGO powder	0,3598	0,1507 to 0,5689	Yes	*
control vs. GO film	-0,03061	-0,2397 to 0,1785	No	ns
control vs. PRGO film	0,6808	0,4614 to 0,9001	Yes	***
control vs. FRGO film	0,7297	0,5104 to 0,9491	Yes	***

Test details	Mean 1	Mean 2	Mean Diff,	SE of diff,	n1	n2	t	DF
control vs. GO powder	1	0,7485	0,2515	0,07473	6	6	3,366	33
control vs. PRGO powder	1	0,7029	0,2971	0,07473	6	6	3,975	33
control vs. FRGO powder	1	0,6402	0,3598	0,07473	6	6	4,814	33
control vs. GO film	1	1,031	-0,03061	0,07473	6	6	0,4097	33
control vs. PRGO film	1	0,3192	0,6808	0,07838	6	6	8,685	33
control vs. FRGO film	1	0,2703	0,7297	0,07838	6	6	9,31	33

Figure 8 B

α -Synuclein

ANOVA summary

Table Analyzed	Data 1
ANOVA summary	
F	12,56
P value	< 0,0001
P value summary	****
Are differences among means statistically significant? (P < 0.05)	Yes
R square	0,8433
Brown-Forsythe test	
F (DFn, DFd)	0,7642 (6, 14)
P value	0,6099
P value summary	ns
Significantly different standard deviations? (P < 0.05)	No
Bartlett's test	
Bartlett's statistic (corrected)	
P value	
P value summary	
Significantly different standard deviations? (P < 0.05)	

ANOVA table	SS	DF	MS	F (DFn, DFd)	P value

Treatment (between columns)	1,411	6	0,2351	F (6, 14) = 12,56	P < 0,0001
Residual (within columns)	0,2622	14	0,01873		
Total	1,673	20			

**Dunnett's multiple comparisons test**	**Mean Diff,**	**95% CI of diff,**	**Significant?**	**Summary**
POSITIVE CON**TROL ROT** vs. GO p**owder ROT**	0,1621	-0,1633 to 0,4876	No	ns
POSITIVE CON**TROL ROT** vs. PRGO p**owder ROT**	0,5231	0,1977 to 0,8485	Yes	**
POSITIVE CON**TROL ROT** vs. FRGO p**owder ROT**	0,7035	0,3781 to 1,029	Yes	***
POSITIVE CON**TROL ROT** vs. GO film **ROT**	0,6384	0,3130 to 0,9639	Yes	***
POSITIVE CON**TROL ROT** vs. PRGO film **ROT**	0,7103	0,3849 to 1,036	Yes	***
POSITIVE CON**TROL ROT** vs. FRGO film **ROT**	0,3368	0,01134 to 0,6622	No	ns

Test details	Mean 1	Mean 2	Mean Diff,	SE of diff,	n1	n2	q	DF
POSITIVE CON**TROL ROT** vs. GO p**owder ROT**	1	0,8379	0,1621	0,1117	3	3	1,451	14
POSITIVE CON**TROL ROT** vs. PRGO p**owder ROT**	1	0,4769	0,5231	0,1117	3	3	4,682	14
POSITIVE CON**TROL ROT** vs. FRGO p**owder ROT**	1	0,2965	0,7035	0,1117	3	3	6,296	14
POSITIVE CON**TROL ROT** vs. GO film **ROT**	1	0,3616	0,6384	0,1117	3	3	5,714	14
POSITIVE CON**TROL ROT** vs. PRGO film **ROT**	1	0,2897	0,7103	0,1117	3	3	6,357	14
POSITIVE CON**TROL ROT** vs. FRGO film **ROT**	1	0,6632	0,3368	0,1117	3	3	3,014	14

Figure 8 C

c-Fos

ANOVA summary

F	7,856
P value	0,0017
P value summary	**
Are differences among means statistically significant? (P < 0.05)	Yes
R square	0,766
Brown-Forsythe test	
F (DFn, DFd)	0,9075 (5, 12)
P value	0,5076
P value summary	ns
Significantly different standard deviations? (P < 0.05)	No

ANOVA table	SS	DF	MS	F (DFn, DFd)	P value
Treatment (between columns)	16,96	5	3,392	F (5, 12) = 7,856	P = 0,0017
Residual (within columns)	5,18	12	0,4317		
Total	22,14	17			

Dunnett's multiple comparisons test	Mean Diff,	95% CI of diff,	Significant?	Summary
con**trol** vs. GO p**owder**	-0,3901	-1,837 to 1,056	No	ns
con**trol** vs. PRGO p**owder**	-2,11	-3,556 to -0,6633	Yes	*
con**trol** vs. FRGO p**owder**	-0,6835	-2,130 to 0,7631	No	ns
con**trol** vs. GO film	-2,037	-3,483 to -0,5901	Yes	*
con**trol** vs. PRGO film	-2,154	-3,600 to -0,7071	Yes	*
con**trol** vs. FRGO film	0,3152	-1,131 to 1,762	No	ns

**Test details**	**Mean 1**	**Mean 2**	**Mean Diff,**	**SE of diff,**	**n1**	**n2**	**q**	**DF**
**control vs. GO powder**	**1**	**1,39**	**-0,3901**	**0,4967**	**3**	**3**	**0,7854**	**14**
**control vs. PRGO powder**	**1**	**3,11**	**-2,11**	**0,4967**	**3**	**3**	**4,248**	**14**
**control vs. FRGO powder**	**1**	**1,683**	**-0,6835**	**0,4967**	**3**	**3**	**1,376**	**14**
**control vs. GO film**	**1**	**3,037**	**-2,037**	**0,4967**	**3**	**3**	**4,101**	**14**
**control vs. PRGO film**	**1**	**3,154**	**-2,154**	**0,4967**	**3**	**3**	**4,336**	**14**
**control vs. FRGO film**	**1**	**0,6848**	**0,3152**	**0,4967**	**3**	**3**	**0,6347**	**14**

Figure 8 D

c-Fos

ANOVA summary	
F	3,45
P value	0,0157
P value summary	*
Are differences among means statistically significant? (P < 0.05)	Yes
R square	0,4964
Brown-Forsythe test	
F (DFn, DFd)	0,8233 (6, 21)
P value	0,5646
P value summary	ns
Significantly different standard deviations? (P < 0.05)	No

ANOVA table	SS	DF	MS	F (DFn, DFd)	P value
Treatment (between columns)	0,8583	6	0,143	F (6, 21) = 3,450	P = 0,0157
Residual (within columns)	0,8708	21	0,04147		
Total	1,729	27			

Dunnett's multiple comparisons test	Mean Diff,	95% CI of diff,	Significant?	Summary
POSITIVE CON**TROL ROT** vs. GO p**owder ROT**	0,05252	-0,3492 to 0,4542	No	ns
POSITIVE CON**TROL ROT** vs. PRGO p**owder ROT**	0,146	-0,2557 to 0,5477	No	ns

POSITIVE CONTROL ROT vs. FRGO powder ROT	0,3267	-0,07499 to 0,7284	No	ns
POSITIVE CONTROL ROT vs. GO film ROT	-0,1312	-0,5329 to 0,2705	No	ns
POSITIVE CONTROL ROT vs. PRGO film ROT	-0,1813	-0,5830 to 0,2204	No	ns
POSITIVE CONTROL ROT vs. FRGO film ROT	-0,1838	-0,5855 to 0,2179	No	ns

Test details	Mean 1	Mean 2	Mean Diff,	SE of diff,	n1	n2	q	DF
POSITIVE CONTROL ROT vs. GO powder ROT	1,136	1,084	0,05252	0,144	4	4	0,3648	21
POSITIVE CONTROL ROT vs. PRGO powder ROT	1,136	0,99	0,146	0,144	4	4	1,014	21
POSITIVE CONTROL ROT vs. FRGO powder ROT	1,136	0,8093	0,3267	0,144	4	4	2,269	21
POSITIVE CONTROL ROT vs. GO film ROT	1,136	1,267	-0,1312	0,144	4	4	0,911	21
POSITIVE CONTROL ROT vs. PRGO film ROT	1,136	1,317	-0,1813	0,144	4	4	1,259	21
POSITIVE CONTROL ROT vs. FRGO film ROT	1,136	1,32	-0,1838	0,144	4	4	1,277	21
