# Supplementary figures and images for: Graphene Oxide and Reduced Derivatives, as Powder or Film Scaffolds, Differentially Promote Dopaminergic Neuron Differentiation and Survival
Source: Front Neurosci. 2020 Dec 21;14:570409. doi: 10.3389/fnins.2020.570409 (PMC7779605; doi:10.3389/fnins.2020.570409)

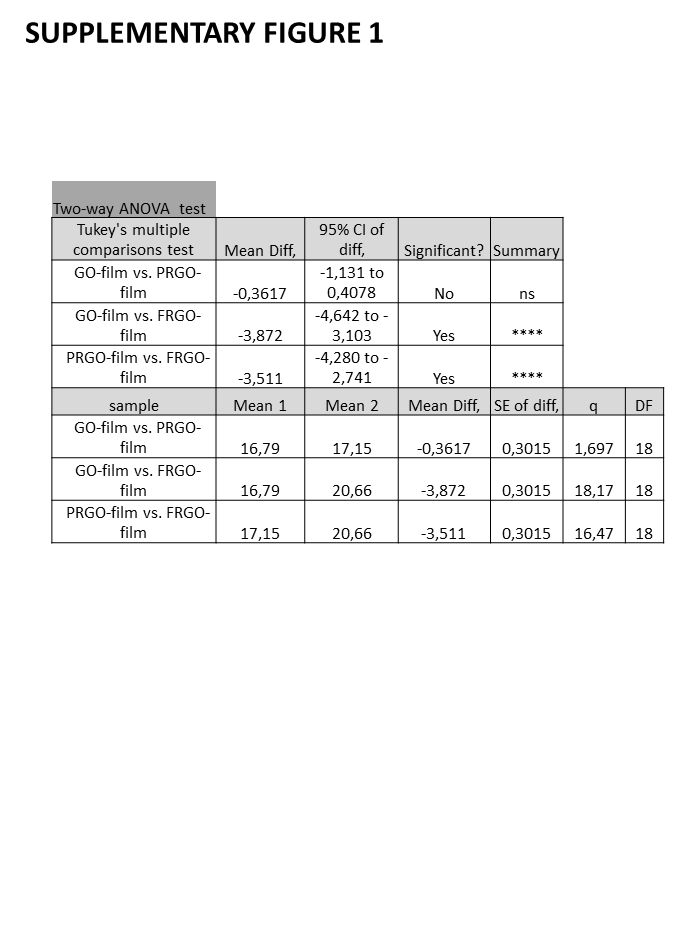

Supplement: Supplementary Figure 1 — Table showing the significant differences in the chemical composition of the films used in the study (Two-way ANOVA, ****p < 0.0001). [file Image_1.TIF]

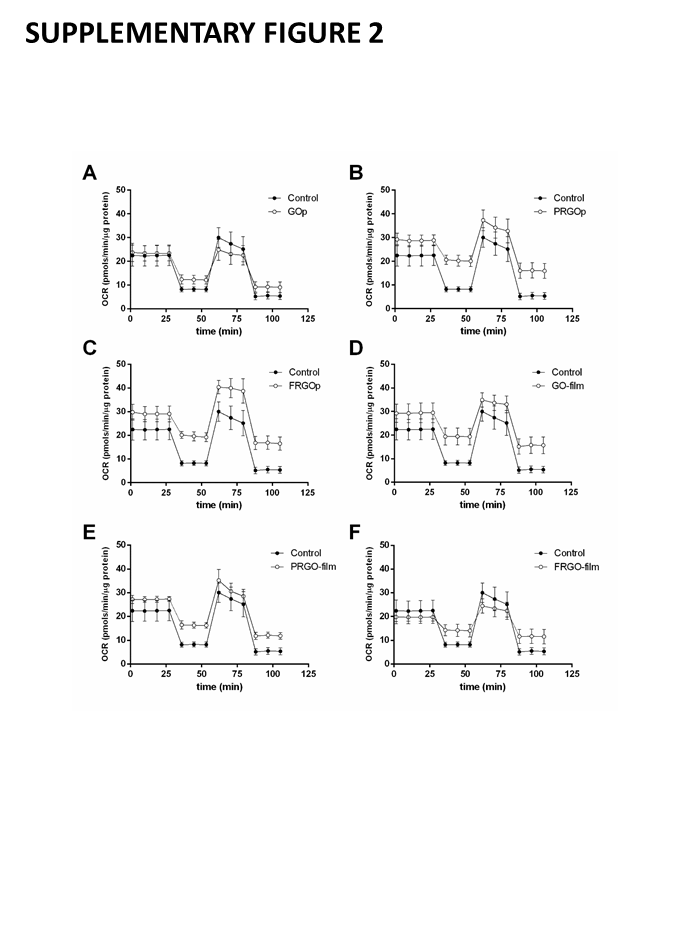

Supplement: Supplementary Figure 2 — Oxygen consumption rate (OCR measure in pmols/min/μg of protein) for GO powder (A); PRGO powder (B); FRGO powder (C); GO film (D); PRGO film (E); FRGO film (F). [file Image_2.TIF]

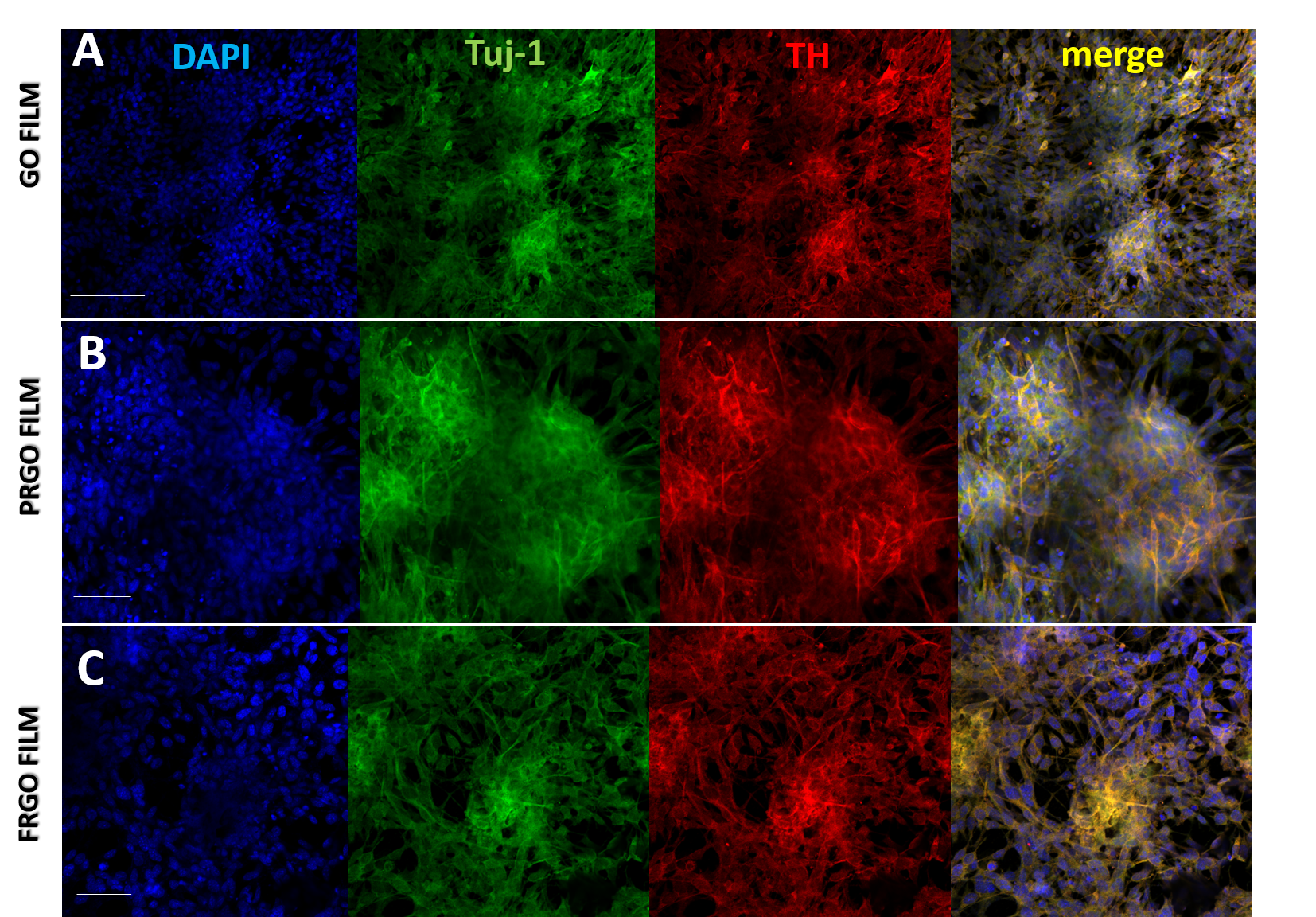

Supplement: Supplementary Figure 3 — Photographs show immunofluorescence of cells stained with DAPI (nuclei marker in blue) Tuj-1/βIII tubulin 1 (green) and TH (red), showing the neuronal process in regions with a high number of positive cells after a long time in culture (4 weeks). (A) GO film (microflakes), scale bar 100 μm; (B) PRGO film (microflakes), scale bar 50 μm; (C) FRGO film (microflakes), scale bar 50 μm. [file Image_3.TIF]

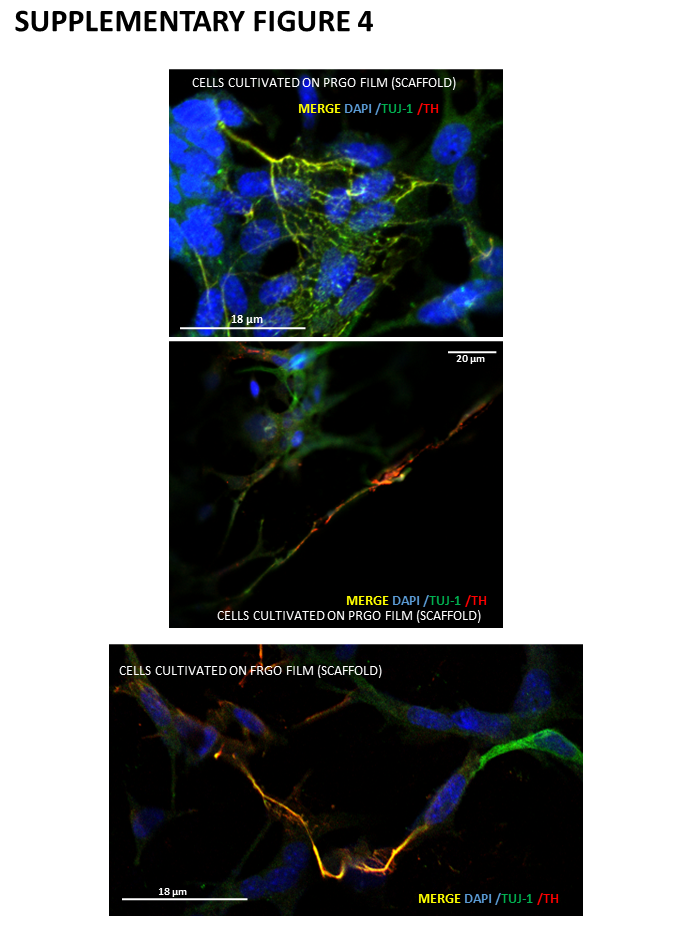

Supplement: Supplementary Figure 4 — Morphological changes of SN4741 cells when cultured on Film scaffolds. Photographs show immunostaining of cells stained with DAPI (nuclei in blue) Tuj-1/βIII tubulin 1 (green) and TH (red), showing neuronal processes. (A) Arborescent neurite-like structures in cells grown on PRGO-film. (B) Contact between TH-positive neurites from cells grown on FRGO film. (C) Cells cultivated on FRGO film. [file Image_4.TIF]

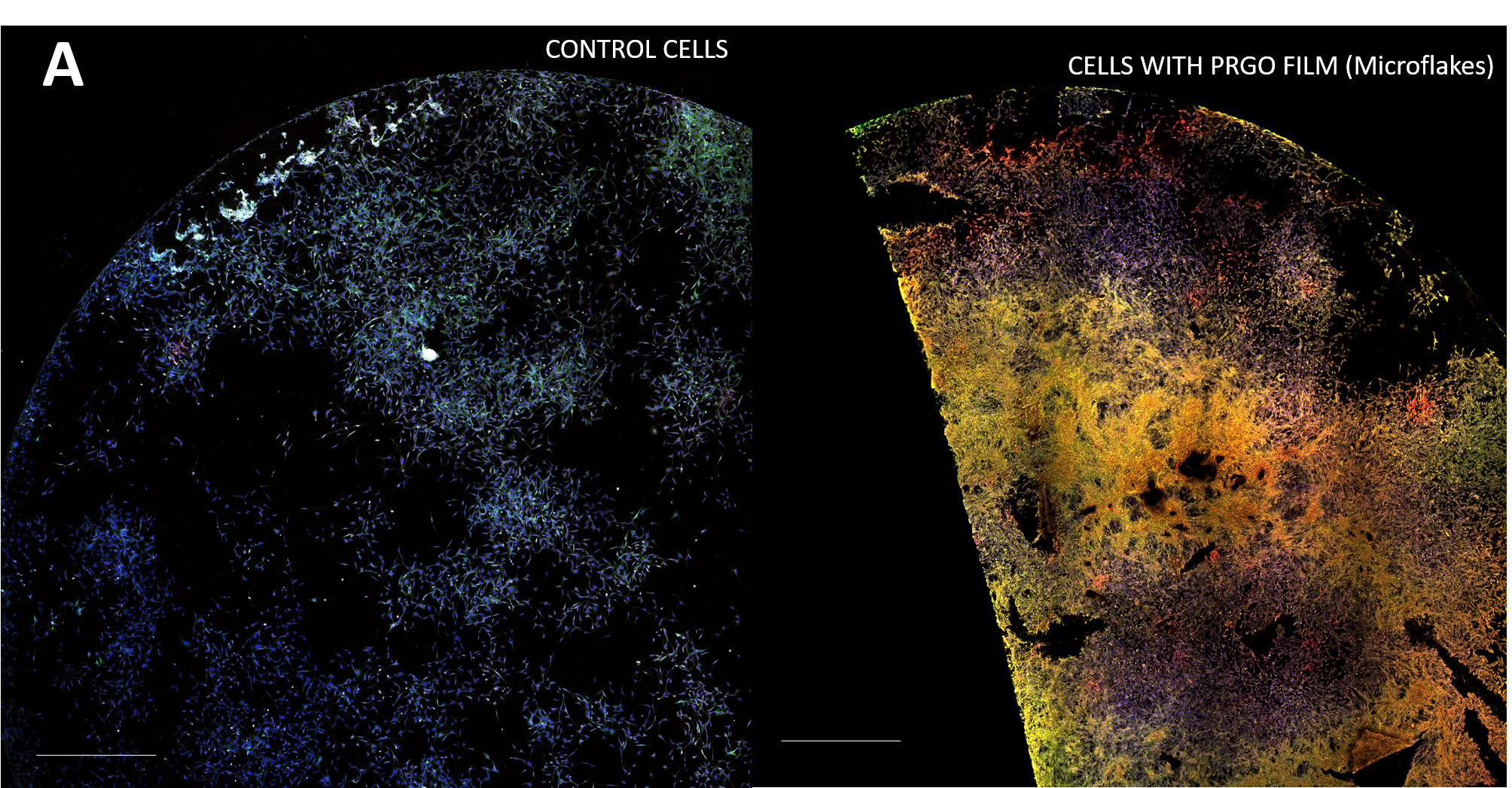

Supplement: Supplementary file 6 [file Image_5.tif]

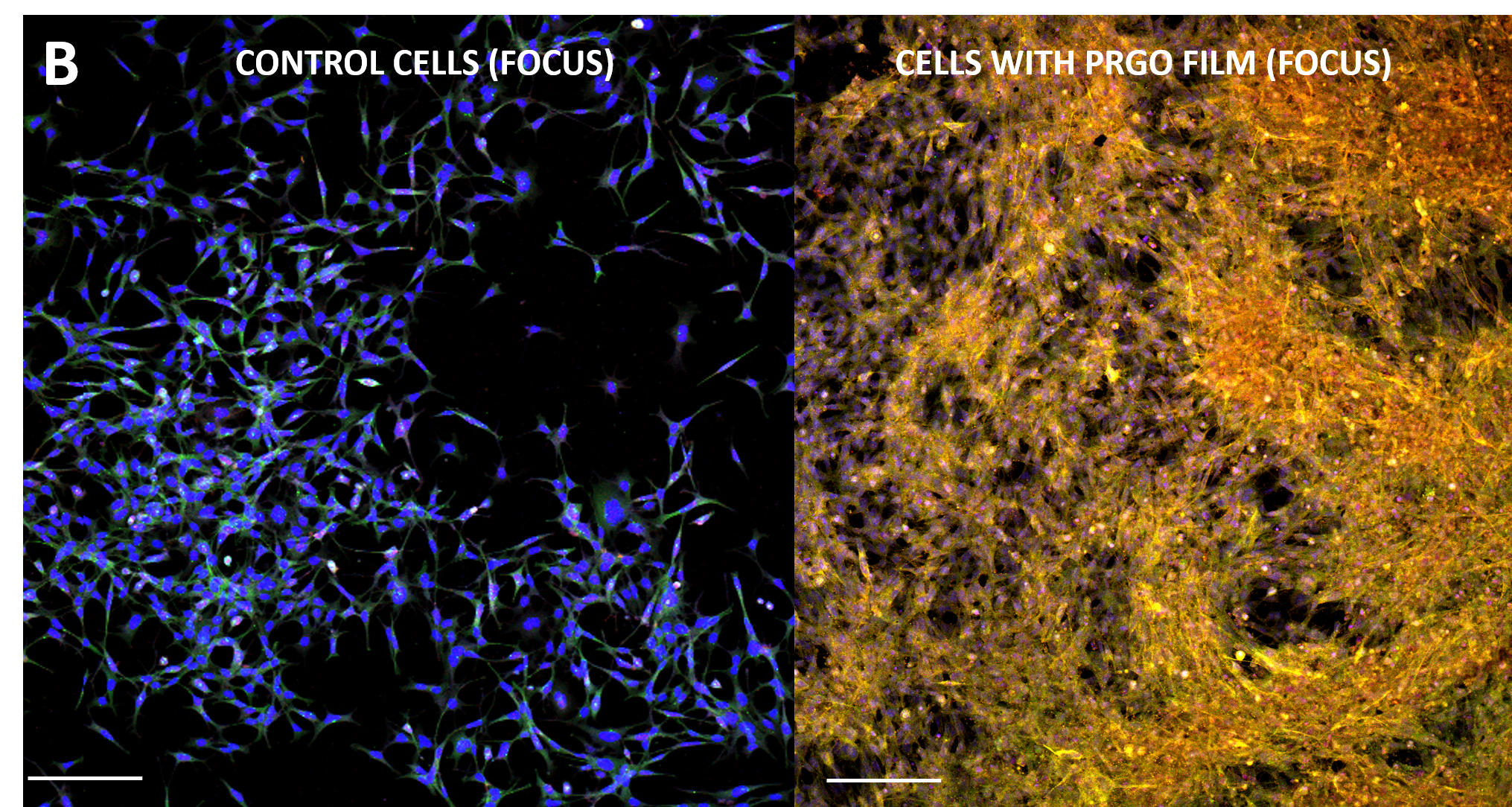

Supplement: Supplementary Figure 5 — Comparison of fragments of 45 mm glass coverslip containing SN4741 cells cultured without (control) for 7 days or with PRGO for 4 weeks. (A) Cells were stained with Dapi (blue), Tuj-1 (green), and TH (red), and photographed at low magnification (scale bar, 1 mm). (B) A detail of part of the coverslip is shown at higher magnification (scale bar, 250 μm). [file Image_6.tif]
